# Breast cancer dormancy is associated with a 4NG1 state and not senescence

**DOI:** 10.1038/s41523-021-00347-0

**Published:** 2021-10-27

**Authors:** Chloé Prunier, Ania Alay, Michiel van Dijk, Kelly L. Ammerlaan, Sharon van Gelderen, Dieuwke L. Marvin, Amina Teunisse, Roderick C. Slieker, Karoly Szuhai, A. G. Jochemsen, Xavier Solé, Peter ten Dijke, Laila Ritsma

**Affiliations:** 1 Department of Cell and Chemical Biology and Oncode Institute, Leiden University Medical Center, 2333 ZC Leiden, The Netherlands; 2 Oncode Institute, Utrecht, The Netherlands; 3 Unit of Bioinformatics for Precision Oncology, Catalan Institute of Oncology, L’Hospitalet de Llobregat, Barcelona, Spain; 4 Molecular Mechanisms and Experimental Therapy in Oncology Program (Oncobell), Bellvitge Biomedical Research Institute (IDIBELL), L’Hospitalet de Llobregat, Barcelona, Spain; 5 CIBER of Epidemiology and Public Health (CIBERESP), Madrid, Spain; 6 Present Address: R&D department, Inovotion SAS, 38700 La Tronche, France

**Keywords:** Metastasis, Senescence

## Abstract

Reactivation of dormant cancer cells can lead to cancer relapse, metastasis, and patient death. Dormancy is a nonproliferative state and is linked to late relapse and death. No targeted therapy is currently available to eliminate dormant cells, highlighting the need for a deeper understanding and reliable models. Here, we thoroughly characterize the dormant D2.OR and ZR-75-1, and proliferative D2A1 breast cancer cell line models in vivo and/or in vitro, and assess if there is overlap between a dormant and a senescent phenotype. We show that D2.OR but not D2A1 cells become dormant in the liver of an immunocompetent model. In vitro, we show that D2.OR and ZR-75-1 cells in response to a 3D environment or serum-free conditions are growth-arrested in G1, of which a subpopulation resides in a 4NG1 state. The dormancy state is reversible and not associated with a senescence phenotype. This will aid future research on breast cancer dormancy.

## Introduction

Breast cancer (BC) is the second most-deadliest cancer in women worldwide^1^. Most BC patients do not succumb to the primary tumor, but rather die from overt metastases that arise later during disease progression. Follow-up analysis showed that 62% of BC patients die 5–20 years after initial treatment due to late relapse (recurrence or metastasis). Late relapse has been linked to a nonproliferative cell state called dormancy^2^. Dormant cells can remain undetected in the tissue for years or decades, until their escape results in proliferation and the formation of recurrent disease or metastasis. In addition, dormancy is at the basis of a drug-tolerance state in certain models, allowing cells to evade apoptosis and survive anticancer therapy^3–5^. As such, dormancy is a major clinical issue as reactivation of dormant cancer cells can lead to cancer relapse and eventually patient death^6,7^.

Several signaling pathways have been described to promote dormancy, such as the transforming growth-factor beta (TGF-β) family^8^, the urokinase receptor uPAR and downstream targets p38α/β and ERK1/2 MAP kinases, the cyclin-dependent kinase inhibitors (CDKN) 1 A (p21) and 1B (p27), integrins^9^, and others reviewed here^8,10,11^. Most of these pathways regulate proteins involved in the cell cycle, and ultimately result in a G0/G1 cell cycle arrest. As a consequence, dormant cells are resistant to chemotherapies that target proliferating cells^4,5^. Moreover, no targeted therapy is currently available in the clinic to eliminate dormant cells, nor are reliable prognostic markers that can predict late relapse. This highlights the need for an even deeper understanding of the signaling pathways that govern the dormancy process.

Senescence, a physiological response to replicative or oncogenic stress in normal cells, shares characteristics with dormancy. Similar to dormancy, senescent cells exit the cell cycle and do not proliferate^12^. Senescence was considered irreversible for decades, but in the last 10 years, reversible senescence named “pseudo-senescence” or “senescence-like” has been observed and documented^13,14^. The mechanisms underlying senescence are quite well defined and a variety of markers have been identified. Senescent cells are often characterized by DNA damage, expression of senescence-activated beta-galactosidase (SA-βGal), loss of Lamin B1, and secretion of a senescence-associated secretory profile (SASP). The program can be induced upon activation of tumor suppressor p53 or upregulation of cyclin-dependent kinase inhibitor CDKN2A (p16)^12^. Interestingly, some dormant cells were shown to have senescence phenotypes like SASP and SA-βGal^3,15^.

To gain insight into signaling pathways that regulate dormancy and to better understand a possible role for a senescence program in dormancy, proper dormancy models are required. The most commonly used model being the D2.OR murine mammary cancer cell line, which is then frequently compared side-by-side to the related D2A1 (D2)-proliferative tumor cell line^9^. Both cell lines were derived from different D2 hyperplastic alveolar nodule mammary tumors^16–18^. In vivo, D2.OR cells disseminate, but remain dormant, whereas D2A1 cells form metastases^17,19,20^. This can be modeled by the Matrigel on Top assay (MoT), in which cells are placed on a thin 3D layer of growth-factor-reduced Matrigel. In this model, the D2.OR cells remain dormant, whereas the D2A1 cells do not remain dormant but proliferate^21^. As the field of dormancy is expanding^22^ and this model is increasingly used in the literature^9,23–26^, we felt the need to thoroughly characterize this D2.OR model, and assess if there is overlap with a senescence phenotype.

## Results

### Unbiased transcriptomic comparison of D2 murine mammary cancer cells

**Fig. 1:**
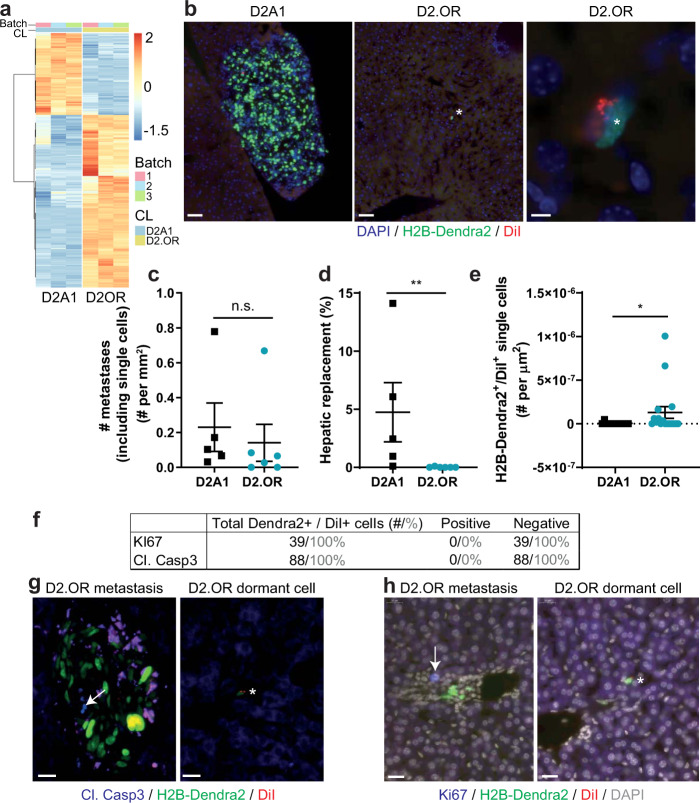
Dormancy of the D2.OR cells in liver parenchyma of syngeneic mice. **a** Heatmap of differentially expressed genes between D2.OR and D2A1 cells cultured in 2D. A total of 1619 genes were selected, based on FC > 1.5, no batch inconsistencies, and a Bonferroni correction for multiple testing >0.05. CL = cell line. Batch = replicate. **b** Microscopy images of D2A1 or D2.OR cell (clusters) in the liver of mice two weeks after intramesenteric vein (i.mes) injection. Scale bar, 50 μm (left and middle) or 5 μm (right). Analysis of **c** metastasis number (including single cells), **d** hepatic replacement by tumor cells, or **e** dormant cells in liver sections of mice sacrificed two weeks after i.mes injection. *n* ≥ 5 mice, three sections per mouse. **f** Quantification (black: number of cells, gray: percentage) of immunostaining on dormant cells in sections with D2.OR cells. **g, h** Representative images of D2.OR metastases and dormant cells (indicated by asterisk) stained for cleaved caspase-3 (**g**) or KI67 (**h**). Staining is indicated in blue (arrow). Scale bars, 20 μm. *N* = 6 mice, three sections per mouse. Indicated *P* values were calculated using Mann–Whitney tests. Error bars, s.e.m.

D2.OR and D2A1 cells are often used to study tumor progression, as they are related tumor-cell lines that differ in their ability to form metastases^27^. As the cells are derived from the same tumor type, but were derived from separately formed tumors in different mice, we wondered how related the cell lines were. We used RNA-seq to assess the differences and commonalities between these cells at the transcriptomic level and identified 1619 differentially expressed genes, of which 522 were downregulated and 1097 were upregulated (Fig. 1a). We performed gene-set enrichment analyses (GSEA) on the gene expression differences between the D2.OR and the D2A1 cell line in 2D culture conditions. Significantly differentially enriched gene sets (FDR < 0.05) were grouped according to their degree of gene overlap (Supplementary Fig. 1). All significantly enriched gene-set categories (e.g., related to phospholipase-C (PLC) activity, cell migration and adhesion, inflammatory response, and embryonic development) were upregulated in the D2.OR cells (i.e., positive Normalized Enrichment Score). Combined, these results suggest that the D2.OR cell line when compared with D2A1 cell line shows upregulation of specific pathways and cellular functions that are mostly related to cell signaling, migration, and development, as well as inflammatory-response processes. A complete list of gene sets tested and their results is available in Supplementary data 1.

### D2.OR murine mammary cancer cells are growth-arrested in the liver

The metastatic (or dormant) capacity of the D2 cells has been assessed using spontaneous and experimental liver and lung-metastasis models^19,23,28^. However, liver metastases were not characterized in an immune-competent model. Thus, we assessed if the D2.OR cells underwent dormancy in fully immune-competent BALB/c mice using an experimental liver metastasis assay. Fluorescently labeled D2 cells were injected into the mesenteric vein and the number and amount of liver metastases (based on fluorescent H2B-Dendra2 signal) was assessed by scoring of fixed–frozen liver sections collected 14 days after implantation (Fig. 1b). Although the same amount of lesions was identified in D2.OR-and D2A1-injected mice (Fig. 1c), the size of the D2.OR lesions was significantly lower compared with the D2A1 lesions, suggesting a growth arrest of D2.OR in vivo (Fig. 1d). We performed label retention using a fluorescent dye to assess dormancy^29^. The injected cells were labeled with a red dye (DiI), which dilutes more or less equally among the two daughter cells upon cell division (Supplementary Fig. 2a). When cell division is reduced by lowering serum levels, DiI is retained (Supplementary Fig. 2b). Thus, single H2B-Dendra2^+^ cells that retained the dye (DiI^+^) did not divide and were considered dormant. Importantly, DiI does not affect cell proliferation in vitro (Supplementary Fig. 2c). We observed very few H2B-Dendra2^+^/DiI^+^ cells in the D2A1 model, and significantly more in the D2.OR model (Fig. 1e). Dormancy of these single D2.OR cells was further validated by the absence of proliferation marker Ki67 and cleaved-caspase3 (CC3) immunostaining, indicating that single H2B-Dendra2^+^/DiI^+^ D2.OR cells are in cell cycle arrest and not apoptotic (Fig. 1f–h). We then studied the in vivo behavior by intravital microscopy. Two weeks after injection, we identified dividing and apoptotic D2A1 cells in large metastases (Supplementary Figs. 3a, b), and label-retaining dormant D2.OR cells (Supplementary Fig. 3c). In three-hour movies, we detected limited D2A1 cell migration (0–3 cells per field of view (~150 cells) moved more than one cell nucleus (six fields of view in two mice)), and no D2.OR cell migration (five cells in two mice, none of which migrated) (Supplementary Figs. 3a and b).

Taken together, the D2A1 cells proliferate and form metastases in the liver of fully competent BALB/c mice, whereas the D2.OR cells do not proliferate and remain in the liver mostly as single dormant cells.

### Dormant D2.OR is in G0/G1 cell cycle arrest

**Fig. 2:**
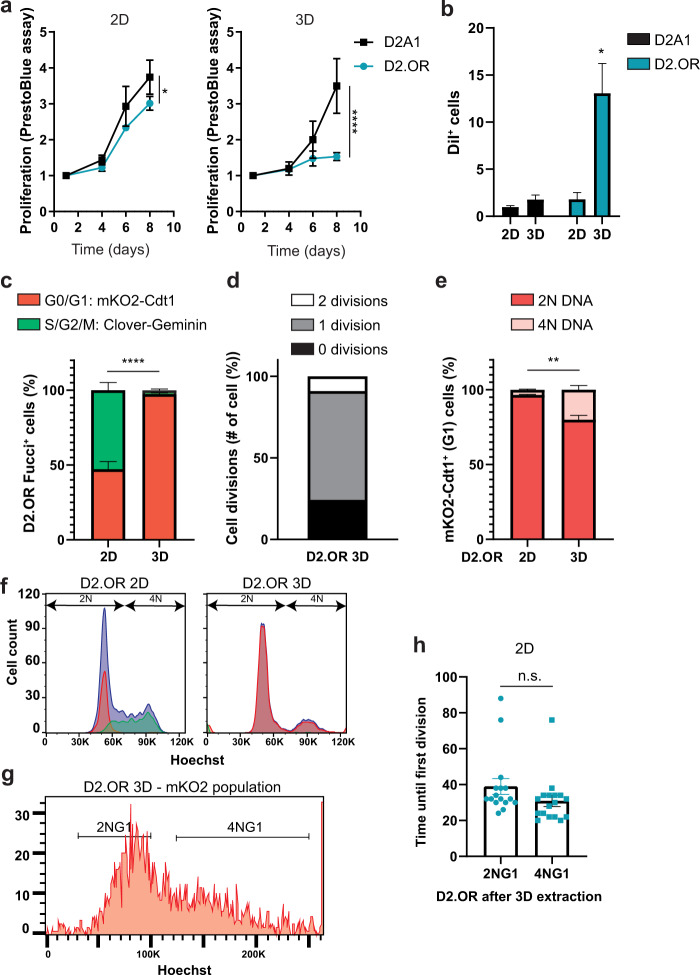
Characterization of D2.OR G1 cell cycle arrest in 3D culture. **a** Proliferation analysis of D2A1 and D2.OR cells in 2D or 3D culture conditions using PrestoBlue cell viability assay. *N* = 3 replicates in duplo/triplo. 2D graph: interaction effect, *F* (3,42) = 3.685, P = *. 3D graph: interaction effect, *F* (3,42) = 10.57, *P* = ****. **b** Quantification of the percentage of DiI^+^ cells after eight days of culture by FACS. *N* = 3 repeats. Interaction effect, *F*(1,6) = 6.278, *P* = *; post hoc test, D2OR 3D vs all others, *P* = *. **c** Quantification of the percentage of Fucci^+^ cells (either Clover^+^ or mKO2^+^) after three days of culture by FACS. *N* = 3 repeats. **d** Quantification of the number of cell divisions until cells remained in G1 (mKO2 + ) after plating in Matrigel. Cells were imaged every 3 h. *N* = 5 positions, 13 cells. **e** Quantification of FACS analysis showing the percentage of mKO2^+^ D2.OR Fucci+ cells containing 2 N or 4 N DNA (measured by Hoechst) in 2D or 3D after three days of culture, *N* = 3. **f** FACS plot of experiment quantified in **c** and **e**. Blue: Hoechst, Green: Clover–Geminin, Red: mKO2–Cdt1. **g** FACS plot of D2.OR-FUCCI4 cells incubated with Hoechst and extracted from 3D and sorted according to strategy as displayed in supplementary Fig. 6c. The mKO2+ cells are shown in a histogram with Hoechst (DNA) on the X axis to show the cell cycle profile. Representative image from *N* = 3. **h** Quantification of the time until the first division of sorted D2.OR cells extracted from 3D Matrigel. The population is based on gates shown in **g**. Quantification of a representative experiment of *N* = 3. *P* values were calculated using a repeated measures 2-way ANOVA or *t*-test. Error bars, s.e.m.

Dormancy is a complex and dynamic process that is more easily studied in vitro. Barkan *et al*. showed that when the D2 cells were cultured in a 3-dimensional assay with low serum and low cell numbers, the D2.OR cells remained dormant, whereas the D2A1 cells proliferated^27^. Several slightly adapted 3-dimensional culture assays have since been used to study the D2 cells in vitro, which all recapitulated the in vivo findings^21,23^. Here, we made use of the (growth-factor-reduced) Matrigel on Top assay (MoT)^21^. Consistent with previous reports, we could show that both the D2.OR and the D2A1 cells proliferate equally in 2D, whereas the D2.OR cells hardly expanded, but the D2A1 cells did expand, in the 3D MoT assay (Fig. 2a and Supplementary Fig. 4a). To assess if the lack in growth expansion in the D2.OR cell in 3D was caused by a growth arrest, or because of a balance between proliferation and death, we determined the percentage of mKi67+ cells and the amount of cleaved caspase-3 (CC3) in the cells in 2D and 3D. CC3 staining was not increased in the D2.OR or D2A1 cells when comparing 3D to 2D (Supplementary Fig. 4b). None of the D2.OR cells in 3D were positive for KI67, in contrast to the cells in 2D and to the D2A1 cells in either growth condition (Supplementary Fig. 4c). These data suggested that a cell cycle arrest was underlying the growth reduction in the D2.OR cells in 3D. To corroborate the D2.OR cell cycle arrest, we loaded dormant D2.OR cells with a DiI and studied its retention or dilution over multiple days. Indeed, DiI was retained in dormant D2.OR compared with the D2A1 cells in 3D, as determined by FACS and live-cell microscopy (Fig. 2b and supplementary Fig. 4d and e).

Dormancy is often defined by a G0/G1 arrest. We made use of the FUCCI cell cycle fluorescent reporter to determine the dormant D2.OR cell cycle status^30^. We engineered D2.OR to stably express fluorescently labeled cell cycle regulators chromatin licensing and DNA-replication factor 1 (Cdt1)_30-120_ and Geminin_1-110_. mKO2- Cdt1_30-120_ is expressed in G1 (red color), both Cdt1–mKO2 and Clover–Geminin_1-110_ are expressed in S (yellow color), and Clover–Geminin_1-110_ is expressed in G2/M phase (green color). After validating the FUCCI reporter system (Supplementary Fig. 5a), we assessed the cell cycle status of D2.OR_Fucci cells in vitro by fluorescence-activated cell sorting (FACS) and microscopy. During 2D proliferation, between 35 and 45% D2.OR cells were in G0/G1, whereas in the MoT, >99% cells were arrested in G0/G1 (Fig. 2c and supplementary Fig. 5b–e). To assess when and if cells divide after plating in MoT, we performed live-cell imaging over 69 h. Interestingly, we observed that immediately after plating, D2.OR cells demonstrated a migratory and stretched phenotype (Supplementary Fig. 5f). When cells made new contacts, they clustered together and thereafter remained in this cluster (Supplementary Fig. 5f and Movie 1). At the end of the observation period, cells showed a rounded phenotype (Supplementary Fig. 5f and Movie 1). We furthermore observed that most cells underwent 1-cell division, although some underwent no or 2-cell divisions (Fig. 2d). The median time it took before all cells in a cluster were in G0/G1 was 24 h, and by day 3, all cells were in G0/G1 (Supplementary Fig. 5g). Altogether, these results confirmed the G0/G1 cell cycle arrest of the D2.OR cells in 3D, and suggest that cells undergo a maximum of two cell cycles after plating in Matrigel.

### Dormant D2.OR can be 4NG1

G1 cells are normally diploid (2 N), however, it has been observed that some G1-arrested cells are tetraploid (4NG1). This G1 arrest is important to prevent tetraploid cells becoming aneuploid^31,32^. We thus wondered what the ploidy of dormant D2.OR cells was. We first performed karyotyping, and concluded that both cell lines are polyploid (Supplementary Fig. 6a). But where D2.OR cells are mostly triploid, D2A1 cells are mostly hypotetraploid. Next, we performed a Hoechst FACS cell cycle analysis on the D2A1 and D2.OR-Fucci cells. For simplicity, we refer to cells that have not replicated their chromosomes yet in S phase to 2 N, despite them being polypoid (Supplementary Fig. 6a) and thus having three or sometimes four copies of the same chromosome. As expected, in 2D, the G1 cell cycle status matches the 2 N state, and the G2 cell cycle status matches the 4 N state (Fig. 2e and f and Supplementary Fig. 6b). Interestingly, in D2.OR cells in 3D, a significant population (~20%) of mKO2 + G1 cells is 4 N (Fig. 2e and f and Supplementary Fig. 6b). Thus, some arrested D2.OR cells reside in 4NG1.

To assess if these D2.OR 4NG1 cells are contributing to the dormant phenotype, i.e., are also able to reenter the cell cycle, we extracted D2.OR-Fucci cells from the Matrigel, FACS-sorted the 4NG1 and 2NG1 cell population (Fig. 2g and Supplementary Fig. 6c), plated them on plastic, and imaged them for five days. For both populations, the time it takes until the first division was around 37 h (Fig. 2h). This suggests that some arrested cells reside in 4NG1, and this population can reenter the cell cycle, defining them as dormant.

### Validation of 4NG1 phenotype in a second-dormancy breast cancer model

To assess the generalizability of this 4NG1 phenotype to other dormant BC models, we made use of a recently published dormancy model using the ZR-75-1 cells^33^. After culturing these cells for at least seven days without serum, the cell population is greatly reduced compared with cells cultured with serum (Supplementary Fig. 7a). Importantly, most serum-starved cells have lost proliferation marker mKI67 (Supplementary Fig. 7b), suggesting a cell cycle arrest. Refeeding the serum-starved cells with FBS results in reversibility of the dormant phenotype (Supplementary Fig. 7c). Under serum conditions, a relatively high proportion (~82%) of the ZR-75-1 cells resides in G1 (Supplementary Fig. 7d). As expected, this increases to ~95% under starving conditions. Interestingly, under normal conditions, the 4N peak consists of mKO2 + (G1), Clover + (G2-M), and double-positive (S) cells (Supplementary Fig. 7e, g). Similarly, of the mKO2 + G1 population, about 21% resides in 4N, making them 4NG1 (Supplementary Fig. 7f–g). Under starving conditions, the 4N peak becomes more mKO2 + (G1) (Supplementary Fig. 7e, g), and the 4NG1 population also increases to ~26% (Supplementary Fig. 7f, g). Thus, ZR-75-1 cells become arrested upon serum starvation, which results in an increased 4NG1 population.

### Migration phenotype alters in Matrigel environment

As migration of the D2.OR cells in the liver is low, whereas D2A1 cells show some migration, we wondered if also these phenotypes recapitulate in vitro. Unexpectedly, D2.OR showed much greater wound healing and speed compared with D2A1 in 2D. In contrast, in a 3D-invasion assay, the D2A1 cells showed greater invasion capacity compared with the D2.OR cells (Supplementary Fig. 8a–e). This suggests that in 3D, but not in 2D, the migration phenotype recapitulates the in vivo migration phenotype.

### Dormant D2.OR genotype does not correlate with senescence signature

**Fig. 3:**
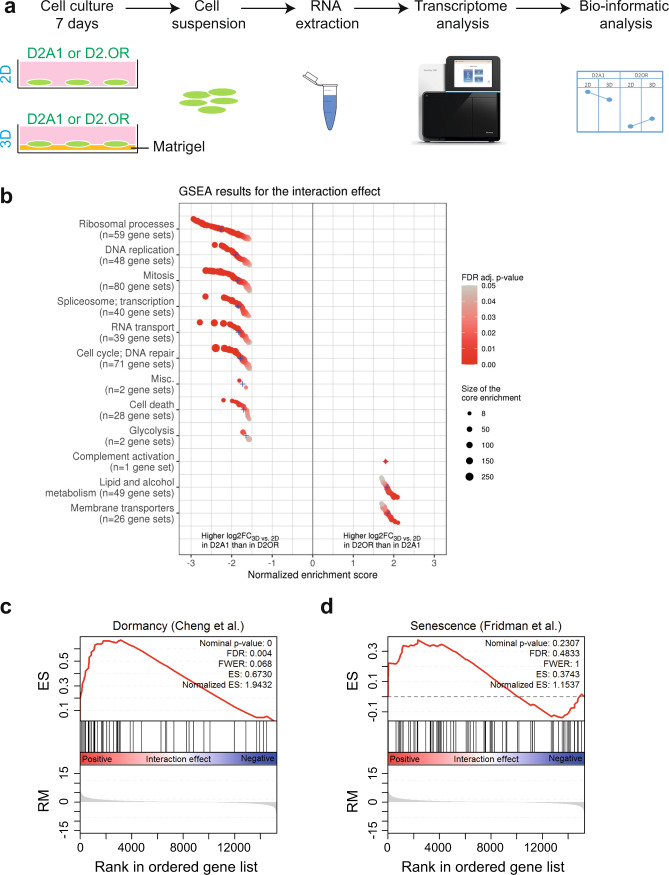
Unbiased transcriptomic analysis suggests a D2.OR dormant cell cycle arrest. **a** Schematic representation of the experimental pipeline. **b** GSEA analysis on the interaction effect when comparing D2A1 and D2.OR in 2D and 3D. Positive normalized enrichment score (NES) indicates that gene sets that were log2-fold change (log2FC) 3D vs. 2D in D2.OR cell line are higher than the log2FC(3D–2D) in D2A1. Respectively, negative NES that corresponds to gene sets was log2FC(3D–2D) that is higher in D2A1 than in D2.OR. Gene sets ordered by normalized enrichment score (NES) within each cluster. Blue crosses represent average NES in each cluster. Point color represents FDR-adjusted *p*-value and size represents core enrichment. GSEA = gene-set enrichment analysis. **c, d** GSEA analysis of a published dormancy signature (Cheng et al.^53^) (**c**) or senescence signature (Fridman et al.^54^) (**d**) on the interaction effect when comparing D2A1 and D2.OR in 2D and 3D. ES enrichment score, RM ranking metric.

Dormancy and senescence are both characterized by cell cycle exit, and dormant-like senescence programs have been postulated^3,15^. As such, we wondered if the D2.OR cells in the MoT model should be considered dormant or senescent. To assess this in an unbiased manner, we performed RNA-seq on the D2.OR and D2A1 cells in 2D and 3D (Fig. 3a) after seven days of culture. We aimed to identify those genes that showed a significant interaction between the cell lines and the culture type (Supplementary Fig. 9a). Once we generated a ranked list of genes based on their statistical significance, we performed a GSEA analysis to identify the enriched pathways at both ends of the list. Gene sets related to proliferation and cell cycle (mitosis) showed a negative normalized enrichment score (NES), i.e., a higher log_2_-fold change (3D–2D) in D2A1 than in D2.OR (Fig. 3b and Supplementary Fig. 9a). Interestingly, similar to our previous study in an unrelated dormancy model, we identified gene sets that associated with the dormancy D2.OR phenotype (i.e., higher log_2_-fold change (3D–2D) in D2.OR than in D2A1) were related to lipid and alcohol metabolism^34^.

Next, we performed preranked GSEA analyses of published dormancy and senescence gene sets using the results of the interaction analysis as the ranking metric. The dormancy signature by Cheng *et al*. was found to be positively enriched (FDR < 0.05) in the D2.OR 3D cells (i.e., higher log_2_-fold change (3D–2D) in D2.OR than in D2A1) (Fig. 3c and Supplementary Fig. 9a). Additionally, we did not observe a significant enrichment for the senescence signature (Fig. 3d).

Last, we performed preranked GSEA analyses of two published SASP gene sets using the results of the interaction analysis as the ranking metric. The Zhang *et al* signature was not significantly enriched^35^, and the signature by Basisty *et al*^36^ was found to be negatively enriched (FDR < 0.002) in the D2.OR 3D cells (i.e., higher log_2_-fold change (3D–2D) in D2A1 than in D2.OR) (Supplementary Fig. 9b).

Thus, gene sets associated with cell cycle progression and SASP were negatively enriched, and dormancy-related gene sets were enriched in our analysis, pointing more toward a dormancy phenotype for the D2.OR cells in 3D. A complete list of gene sets tested and their results is available in Supplementary data 2.

### Pathways involved in senescence are not active in dormant D2.OR

Molecularly, dormancy is a different process from senescence. Senescence is not defined by specific markers, but rather by a multimarker approach^37^. To determine if a senescence program might be responsible for the observed cell cycle arrest, we assessed the following senescence-associated markers: SA-β-galactosidase, DNA damage (γ-H2AX), and the loss of Lamin B1.

**Fig. 4:**
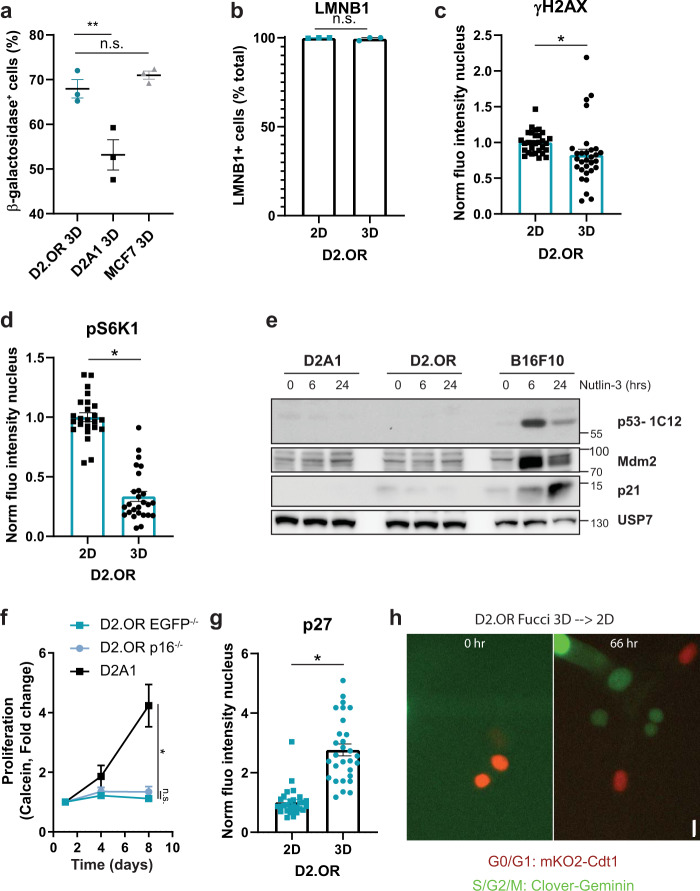
D2.OR cell cycle arrest in 3D is associated with dormancy and not senescence. **a** Quantification of the percentage of β-galactosidase+ cells cultured for four days in 3D. MCF7 was used as a senescence control. *N* = 3 replicates. **b** Quantification of the percentage of LMNB1 + cells by immunofluorescence cultured for four days in 2D or 3D conditions. *N* = 3 replicates, each ≥12 cells. **c** Quantification of immunofluorescence nuclear γH2AX staining intensity of D2.OR cells cultured for four days. Replicates normalized to 2D. *N* = 30 field of view (FOV) of three replicates. **d** Quantification of pS6K1 immunostaining of D2.OR cells cultured in 2D or 3D. Replicates are normalized to 2D. *N* ≥ 24 field of view (FOV) of three replicates. **e** Immunoblot analysis of lysates from indicated cell lines cultured in 2D and treated with Nutlin-3 [10 μM] for 0, 6, or 24 h. **f** Quantification of proliferation of indicated cell lines cultured for eight days in 3D. Replicates were normalized to day 1. *N* = 3 replicates, in duplo. Time × Cell line, *F*(4, 27) = 10.68. *P* = ****. Post hoc test, day 8, D2A1 vs others *P* = *, D2.OR EGFP vs P16-/- *P* = n.s. **g** Immunofluorescence quantification of the normalized fluorescence intensity in the nucleus of D2.OR cells grown for five days in 2D or 3D. *N* = 3 replicates, each 10 FOV. **h** Microscopy images of a time series of D2.OR Fucci cells in 2D. Cells were cultured in 3D for four days, extracted, replated in 2D, and imaged. Scalebar, 30 μm. *P* values were calculated using 1-way ANOVA, nested *T*-test or mixed-effect analysis. Error bars, s.e.m.

We observed that dormant D2.OR expressed β-galactosidase as much as the senescence control (MCF7 treated with etoposide in 2D), whereas the proliferative control (D2A1) did not (Fig. 4a). In contrast, Lamin B1 loss was not observed in dormant D2.OR, whereas it was lost in a senescence control (irradiated MCF7 cells) (Fig. 4b and Supplementary Fig. 10a). Senescent cells are known to accumulate DNA damage. We therefore checked DNA damage through γ-H2AX staining. Upon DNA double-stranded breaks, γ-H2AX gets phosphorylated and accumulates in the cell forming small foci. Interestingly, we did not find an increase of foci accumulation in dormant D2.OR compared with control (D2.OR 2D), whereas foci did accumulate in the senescence control (HeLa cells treated with etoposide) (Fig. 4c and Supplementary Fig. 10b). Last, the results in the D2A1 cells were comparable to the D2.OR cells (Supplementary Fig. 10).

Dormancy is often associated with low levels of mammalian target of rapamycin (mTOR) (e.g., due to low nutrient levels), whereas during senescence, mTOR levels are often high^37–39^. We therefore assessed the activity of mTOR by performing immunofluorescence of downstream kinase target S6K1. Phosphorylation of S6K1 suggests higher mTOR activity. D2.OR cells plated in 3D had significantly lower p-S6K1 levels compared with D2.OR cells plated in 2D. As expected, senescent cells (Hela cells treated with etoposide) did not show a reduction in p-S6K1 (Fig. 4d and supplementary Fig. 10c). Interestingly, in the D2A1 cells in 3D, the p-S6K1 levels were also decreased compared with 2D (Supplementary Fig. 10). This indicates that p-S6K1 can merely be used as a senescence marker, and not a dormancy marker in our model. As three out of the four senescence markers were not present in our phenotype, this argues against a senescence phenotype.

p53 and p16 are the main pathways that can induce senescence, thus, we wanted to investigate if p53 and/or p16 would be required for the observed cell cycle exit in D2.OR cells in 3D. When we assessed the response of both cell lines to Nutlin-3, which stabilizes p53 protein levels, resulting in an increase in downstream target-gene expression, neither of the D2 cell lines showed an increase in mRNA levels of the two p53 target genes, p21 and MDM2, while these genes were increased in a positive-control cell line (B16F10) (Supplementary Fig. 11a). Similar results were observed at the protein level (Fig. 4e). Moreover, we were unable to detect p53 protein in either the D2.OR or D2A1 cell line (Fig. 4e). Combined, the data suggest that neither the D2.OR cells nor the D2A1 cells contain functional p53. P16, however, was expressed by D2.OR cells (Supplementary Fig. 11b). But CRISPR–Cas9-mediated knockout of the *p16* gene did not result in escape from dormancy in the MoT assay (Fig. 4f). These results indicated that the two main senescence-inducing pathways p53 and p16 were not responsible for the D2.OR cell cycle arrest phenotype.

Next, we tested if dormancy markers were associated with the observed cell cycle arrest. Cell cycle inhibitor p27 is often associated with dormancy. p27 showed a specific increase at the protein level in the D2.OR cell line and not the D2A1 line when comparing 2D versus 3D (Fig. 4g and Supplementary Fig. 11c), suggesting a dormancy program. Another hallmark for dormancy is the reversibility of the cell cycle arrest. Even though reversible senescence has been observed, it is not that common, and it has only been observed for cells that have just initiated a senescent program^40^. Thus, we assessed if arrested D2.OR was able to escape from dormancy and start proliferating (assessed by Fucci, or by PrestoBlue cell-viability assay) when cells were recovered from Matrigel and replated in 2D. Indeed, we observed that the dormancy phenotype was reversible (Fig. 4h, Supplementary Fig. 11d and Movie 2).

To assess the generalizability of our results to other dormant BC models, we assessed the senescence and dormancy markers in the ZR-75-1 dormancy model. Indeed, upon assessing the dormancy and senescence markers in this model, also, here we can conclude that the ZR-75-1 cells are in dormancy and not senescence (Supplementary Figs. 10, 11).

Last, we also assessed some of the dormancy/senescence markers on our dormant cells in vivo using immunohistochemistry. Similar to the in vitro findings, senescence markers (gH2AX and LaminB1 loss) were absent (Supplementary Fig. 12).

Combined, we conclude that the cell cycle exit phenotype of dormant D2.OR and ZR-75-1 cells is not associated with a senescence phenotype, but rather with a reversible dormant phenotype.

## Discussion

Dormancy is incompletely understood, but its importance in cancer progression and resistance is evident as reactivation of dormant cancer cells can lead to cancer relapse and eventually patient death^6,7^. Proper dormancy models and a thorough understanding of these models are essential to expose the molecular and cellular characteristics of dormancy. Such models should be specific for dormancy, and should not model other cell cycle arrest processes like senescence. Here, we evaluated the widely used D2 breast cancer cell line model for its capacity to specifically recapitulate dormancy in vitro and in vivo.

Similar to others, we were able to show that the D2.OR cell line underwent a cell cycle arrest in an experimental metastasis model. However, we also show that D2.OR become dormant in the liver of syngeneic mice, i.e., mice with a fully competent immune system. This is important as the adaptive immune system has been shown to regulate single-cell dormancy^23^. Moreover, by combining label retention and immunohistochemistry, we were able to specifically address proliferation and death in the truly dormant cell population. Indeed, all label-retaining cells were negative for KI67 and cleaved caspase-3, excluding “balanced dormancy”, which is based on equal number of proliferating and dying cells^11^. Interesting is that not all D2.OR cells remain dormant in the liver over the course of two weeks. This heterogeneity may be caused by the microenvironment, e.g., through variations in stiffness, or alternatively by cellular heterogeneity. The latter is emphasized by the genomic instability shown as variation in chromosomal numbers and rearrangements we observed between the different metaphase cell-derived nuclei from the same cell line.

Using intravital microscopy, we imaged five dormant D2.OR cells in the liver, none of which migrated. Also, D2A1 showed little migratory activity, as only a few cells per field of view moved. In an in vitro 3D environment, the D2.OR is much less migratory compared with the D2A1 cells, whereas on plastic, the D2.OR cells migrate faster than the D2A1 cells. This suggests that the 3D environment reduces the capacity of the D2.OR cells to migrate. This might be either indirect, as a consequence of the dormancy phenotype, or the dormancy phenotype is a result of the inhibited migratory capacity. As the D2.OR cells were able to migrate in the 3D matrix shortly after plating, we expect the former. In the brain, motile MDA-MB-435-dormant melanoma and nonmotile PC14–PE6-dormant lung carcinoma cells have been observed using intravital microscopy^41^, suggesting that it might be cell-line or tumor-type specific whether dormant cells migrate or not.

The identification of 4NG1 D2.OR cells when cultured in 3D is surprising. So far, this has never been associated with cells in dormancy, but rather with senescence^42^. 4NG1 cells can appear if cells are delayed in mitosis (D-mitosis). In the presence of an active spindle-assembly checkpoint, cells in D-mitosis can ultimately escape (termed mitotic adaptation or slippage) and enter the next G1 as tetraploid cells^42^. These 4NG1 are either arrested in senescence, or are able to proliferate normally. We, on the other hand, suggest that the 4NG1 D2.OR cells cultured in 3D are in dormancy, as they can reenter the cell cycle after plating in a 2D environment. Future research should determine if the 4NG1 cells are present in vivo and in dormancy models of other tumor types as well. At last, it will be of interest to determine the reason for the 4NG1 arrest. Pharmacologic inhibition of CDK4 and CDK6 can induce tetraploidy in some breast cancer tumor models^43^. As such, it is of interest to determine the expression levels of these CDKs in our dormancy models. Alternatively, studies have shown that the absence of p53 protein can accelerate the exit from D-mitosis^44^. As the D2.OR cells are p53 negative, this might explain the presence of 4NG1 cells. However, why the cells were in D-mitosis is unclear, as this is normally induced by treatments that depress or altered microtubule assembly/dynamics (e.g., aurora-kinase inhibitors)^44^.

We showed that the D2.OR cells are undergoing a dormant cell cycle arrest. Why do some of the arrested D2.OR cells in 3D show increased expression of β-galactosidase? It is important to note that none of the senescence markers are specific, and are in fact also expressed by other cells under certain circumstances. Indeed, β-galactosidase expression has been reported in other dormant cells^13^. Thus, senescence can only be confirmed when a multitude of markers is present^37^, which was not the case for the D2.OR cells in 3D.

Many of the senescence markers can be used to identify senescence (as is clear from our senescence-control experiments), however, the absence of these markers does not define dormancy. This is evident when assessing D2A1 cells in 3D. These cells are not dormant, but also show absence of many of the senescence markers. As such, to define if cells are in dormancy, it is important to also show the presence of dormancy markers. Indeed, next to the absence of proliferation marker KI67, we were able to show that the dormant cells reside in G1, and that they upregulate p27. Indeed, p27 is a widely used marker to detect cells in dormancy. Moreover, another “hallmark” of dormancy is the reversible cell cycle arrest, which we were able to show for the D2.OR and ZR-75-1 model. Based on transcriptomic analyses, we show that the D2.OR-dormancy phenotype is associated with gene sets related to lipid and alcohol metabolism. When dormant cells in a completely unrelated 2D assay were assessed, a similar association was found, and this was linked to exosome secretion by dormant cells^34^. This suggests that exosome secretion might be a general characteristic of dormant cells, but it has to be confirmed in the D2.OR model system.

The discrimination between a senescence or dormancy cell cycle arrest program is important when considering treatment options. As the pathways that define either program are quite distinct^39^, targeted treatment for one program will most likely not work against the other program. For example, FOXO4–DRI senolytic eliminates senescent cells by inhibiting the interaction between FOXO4 and p53. As p53 is not expressed in D2.OR-dormant cells, this senolytic will most likely not eliminate these cells. Which drugs will eliminate dormant cells is the subject of active investigation, and can be pursued with the D2.OR dormant-model system.

## Methods

### Cell culture

Mouse mammary carcinoma cell lines D2A1 and D2.OR were obtained from Karmanos Cancer Institute (F.R. Miller)^16^. Mammary cancer cells D2.OR and D2A1, mouse melanoma cancer cell B16F10, human breast cancer cell MCF-7, human cancer cell Hela, and human kidney cell HEK293T were all cultured in Dulbecco’s Modified Eagle Medium (DMEM) high glucose (Gibco), supplemented with 10% fetal bovine serum (FBS) (Biowest), 60 μg/mL penicillin (Roth), and 96 μg/mL streptomycin (Roth). ZR-75-1 cells were cultured in RPMI medium 1640 + L-Glutamine (Gibco, 21875-034), supplemented with 10% fetal bovine serum (Biowest), 60 μg/mL penicillin (Roth), and 96 μg/mL streptomycin (Roth). Cells were maintained in a 5% CO2-humidified incubator (Forma Scientific) at 37 °C. Every month, all cell lines in culture were subjected to a mycoplasma-infection test.

For the standard two-dimensional (2D) experiments, cell density was determined using the TC20™ Automated Cell Counter (Bio-Rad). Thereafter, 1 × 10^3^ cells were seeded per well in either a 48-well or 8-chamber format in DMEM high glucose (Gibco) and refreshed every 3–4 days.

The Fucci reporter construct (pLL3.7m-Clover–Geminin(1–110)–IRES–mKO2–Cdt(30–120)) was a gift from Michael Lin (Addgene plasmid #83841)^30^. Stable Fucci reporter expression and H2B-Dendra2 expression was performed by lentiviral particle transduction. Transfection of HEK293T cells by polyethylenimine (PEI) with a viral vector and helper constructs was performed in 10-cm culture dishes. About 6 mL of virus-containing medium was filtered and supplemented with 10 μg/mL polybrene. The suspension was used for the infection. pLV_CMV_H2B-dendra2 cell selection was performed with medium containing 1.5 μg/mL puromycin, whereas the selection of Fucci was performed by flow cytometry.

### Dormancy assays

For the Matrigel-on-top culture assay^45^, a 48-well plate was coated with 40uL/well of (growth-factor-reduced) Matrigel (Corning) and incubated at 37 °C for 30 min, which allowed the Matrigel to solidify. Subsequently, cells (1000 cells/well) were resuspended as single cells in 200uL of 3D media (Nutrient Mixture F-12 (DMEM/F-12) + GlutaMAXTM-I (Gibco) supplemented with 2% Horse serum (Gibco), 0.5 μg/mL hydrocortisone (Sigma Aldrich, H4001), 50 ng/mL cholera toxin (Sigma Aldrich, C8052), 10 μg/mL insulin (Sigma Aldrich, I3536), 60 μg/mL penicillin (Roth), and 96 μg/mL streptomycin (Roth)) containing 2% Matrigel, and added on top of the solidified Matrigel. For the 2D culture, cells were seeded directly on plastic in the 3D media. Medium was refreshed every 3–4 days.

For the ZR-75-1 assay, 11.000 cells were plated in full serum per 48-well. The next day, the cells were, if indicated, serum-starved for six days. Where indicated, the cells were then washed and plated in full serum until day 11. The medium was refreshed every 3–4 days.

### Cell-viability assay

To determine the proliferation rate in 2D or 3D, cells were cultured for ≤8 days in a 48-well plate as described in the “Dormancy assay” section. At the desired timepoints, 20 μL of PrestoBlue cell-viability reagent (Invitrogen) was added directly to the media and the plate was incubated for 10 min at 37 °C in the dark. Subsequently, fluorescence at wavelength 544/590 nm was captured using the VICTOR X3 plate reader (PerkinElmer). Measurements were normalized to controls with only Matrigel and/or media to adjust for background signals.

Calcein-AM was additionally used to determine proliferation^46^. In short, cells were washed twice with phosphate-buffed saline (PBS) after which 5 µM calcein-AM (Thermofisher, C1430) in PBS was added. After incubating for 30 min at 37 °C in the dark, fluorescence was captured at wavelength 494/517 nm using the VICTOR X3 plate reader (PerkinElmer). Afterward, measurements were normalized to controls with only Matrigel and/or media to adjust for background signals.

### DiI loading

Cells cultured in 2D were trypsinized and counted. In total, 1 × 10^6^ cells/mL were incubated for 7 min with 5uL/mL DiI vibrant solution. Loaded cells were spun down for 3 min at 1300 rpm and the pellet was washed with PBS 2 times. Cells were counted again before plating in 2D or 3D condition. For animal experiment, cells were always loaded with DiI one day prior to the injection.

### FACS

For the 2D condition, D2.OR-FUCCI4 or ZR-75-1-FUCCI4 cells were collected and diluted to 1 × 10^6^ cells/mL suspension in 0.2% BSA in PBS. Thereafter, 2 × 10^6^ cells were incubated with Hoechst 33342 (1 mg/mL) (Thermofisher Scientific, 62249) to stain the nuclei. After incubation in the dark for 30 min at 37 °C, cells were collected in 200–500 μL of PBS and subjected to flow cytometry.

For the 3D condition, D2.OR-FUCCI4 reporter cells were seeded in 6-cm dishes with Matrigel, cultured for four days, and directly stained with Hoechst 33342 (1 mg/mL) (Thermofisher Scientific, 62249) for 30 min at 37 °C. Thereafter, cells were extracted from Matrigel according to our self-optimized protocol in which every step is performed on ice and all tips/pipettes/tubes are precoated with 2,5% BSA (Sigma Aldrich) in PBS to yield the highest number of living cells. The cells were washed two times with PBS, ice-cold Cell Recovery Solution (Corning) was added, and cells were gently rocked 20–30 min on ice in order to dissolve the Matrigel. After dissolvement was confirmed by microscopy, 1% BSA (Sigma Aldrich) was added to the cell suspension and cells were centrifuged (twice) (approximately 280 g at 4 °C) for 5 min. After discarding the supernatant, the cells were resuspended in 200–500 μL of 0.5% BSA (Sigma Aldrich) in PBS and subjected to flow cytometry.

Flow cytometry was performed using the BD LSR II machine and BD FACSDiva software, and sorts were performed on BD FACSAria machines. Afterward, recordings were analyzed using the FlowJo software.

### Immunofluorescence

Cells were cultured on 48-well plates (CELLSTAR), µ-Slide 8 Well (Ibidi), or Nunc Lab-Tek chamber slides (ThermoFisher Scientific), where indicated coated with Matrigel. Cells were fixed in 2% paraformaldehyde for 10 min and permeabilized in PBS supplemented with 0.5% Triton X-100 for 10 min at room temperature (RT). Then cells were washed two times in PBS for 10 min and incubated with blocking buffer (130 mM NaCl, 7,7 mM NaN3, 0.1% bovine serum albumin, 0.2% Triton X-100, 0.05% Tween 20, and 5% goat serum) for 30 min–1h at RT. Primary antibodies were diluted in 0.5x blocking buffer at the following concentrations: Ki67 (ab16667, AbCam, 1:200), Cleaved-Caspase3 (9661, Cell Signaling, 1:200), Anti-phospho-Histone H2A.X (Ser139) (clone JBW301, 05-636 Millipore, 1:1000), p-p70 S6 kinase a (pS6K1)(Santa Cruz, sc-8416, 1:100), LMNB1 (Abcam, ab16048, 1:500), and p27 (3698, CST, 1:800), and incubated overnight at 4 °C or for 1 h at room temperature. Subsequently, cells were washed two times 10 min with PBS and incubated with A488-conjugated secondary antibody diluted in blocking buffer at 1/200 for 1 h at RT. Finally, cells were mounted in media containing DAPI (Hard vectashield set) and immediately analyzed using the Leica SP5 confocal microscope or stored at −20C to prevent Matrigel dissolution and fluorescent-signal degradation.

### Microscopy

Confocal imaging was conducted on the Leica SP5. Images were acquired as sequential scans and collected in 8- or 12-bit. Plastic 48-well plates were imaged using the dry 40x long-distance magnification, whereas the glass 8-well chamber slides were imaged using the 63x oil objectives. The fluorescent-detection range was set manually based on the emission spectra of the different fluorophores. We used the following lasers depending on the fluorophores: 405 Diode, Argon, DPSS561, HeNe594, and HeNe633. Furthermore, Z-stacks differed between 1,5 µm and 3,0 µm, depending on the protein of interest.

Live-cell imaging was conducted on the Leica AF6000 and Leica DMI6000. For both, plastic 48-well plates were imaged using the dry HPX PL FLUOTAR L 40 × 0.6-NA long-distance objective. The following filter cubes were used: geminin-Clover, Triple filter G, cdt1-mk02, RFP, and Brightfield: empty. Live-cell imaging experiments with FUCCI4-expressing D2.OR and D2A1 cells were performed for 1–5 days with frames every 20 min–3 h. Z-stacks were adjusted for individual positions and varied between 2 and 3 µm. Live-cell imaging of D2.OR and D2.A1 cells loaded with DiI was performed for 93 h with frames every 30 min. Furthermore, temperature and CO2 percentage, at respectively 37 °C and 5%, were maintained using an environmental-control system.

Images were gathered with confocal and live-cell imaging analyzed using ImageJ software. To determine mean fluorescence intensity in the nucleus, an ImageJ macro was used for automatic analysis. In short, a mask was created from the DAPI channel by using MaxEntropy (3D) or Huang (2D)-based autothresholding. The analysis channel was multiplied by the mask and then the mean fluorescence intensity in the mask was determined. The same was done for the DAPI channel. In 3D, a DAPI-based single plane from a Z stack was analyzed, in 2D, a single plane as recorded was analyzed. In 3D, we normalized the signal toward the average DAPI signal in 2D, as a positive correlation was observed between signal intensity in DAPI and the measurement channel was observed.

### Immunoblotting

Cells were lysed in Laemmli buffer (12% 1 M Tris-Cl pH 6.8, 4% SDS, 20% glycerol, and ddH2O) supplemented with 1x phosphatase-inhibitor cocktail (P5726, Sigma), and total protein concentration was determined using the DC protein assay (Bio-Rad). In total, 10 µg of protein was loaded per lane. The proteins were separated by 10% sodium dodecyl sulfate polyacrylamide gel electrophoresis and transferred onto a polyvinylidene difluoride membrane using electrophoresis for 1 h. Following, the membrane was blocked at room temperature for 1 h in tris-buffered saline containing Tween-20 (TBS-T) containing 5% milk powder. The membrane was washed with TBS-T and subsequently incubated with the primary antibodies at 4 °C overnight. The types of primary antibodies used were Anti-CDKN2A/p16INK4a ([EPR20418] (Abcam, ab211542)), Anti-Vinculin (hVIN-1 (Sigma Aldrich, V9131, 1:1000)), anti-USP7 (Bethyl Laboratories, A300-033A), anti-MDM2, clone 3G9 (Merck Millipore, 04-1530), anti-p53 clone 1C12 (Cell Signaling Technology), and anti-p21, Clone F5 (Santa Cruz Biotechnology). After washing with TBS-T followed with incubation with horseradish peroxidase (HRP)-conjugated anti-rabbit, anti-goat, or anti-mouse secondary antibody (Amersham Science, 1:10.000) at room temperature for 2 h. Bands were detected using enhanced chemiluminescence (Clarity ECL western blotting substrate, 1705061, Bio-Rad) in accordance with the supplier’s protocol. Full western blots have been supplied in the supplementary figs. All blots are derived from the same experiment and were processed in parallel.

### qPCR

Total RNA of cells was extracted using the Macherey–Nagel RNA kit (Thermo Fisher Scientific, Netherlands). cDNA was made from 1000 ng of RNA mixed with 5x reaction buffer, Rnase inhibitor, and 10 mM dNTP (ThermoFisher, Netherlands) using the Thermo Fisher Scientific PCR protocol, 5 min at 25 °C, 60 min at 42 °C, and 5 min at 70 °C. PCR was performed on the T100 Thermal Cycler (Bio-Rad Laboratories, USA).

cDNA was diluted to 100 ng/μL. A mastermix of RNase-free mQ, forward primer, and reverse primers was added to cDNA along with SybrGreen (ThermoFisher, USA). Gene expression was measured with the BioRad CFX Man 3 (Bio-Rad laboratories, CA, USA). The measured gene expression was corrected with the geomean of *Hmbs* and *Rpl13a*.

### β-galactosidase assay

Cells were seeded 1 × 10^3^ cells/well in a µ-Slide 8 Well (Ibidi, 80826) chambered coverslip in both 2D and 3D. After culturing for four days, cells were washed twice with PBS. Thereafter, the β-galactosidase assay was performed using the Senescence beta-Galactosidase Staining Kit (Cell Signaling, 9860 S) according to the supplied protocol.

Cells were fixed with 1X fixative solution for 15 min at RT, washed with PBS, and stained with 1X β-galactosidase staining solution at pH 6.0. After staining, the chambered coverslip (Ibidi) was stored in a culture dish (Cellstar), sealed with parafilm, and incubated in a dry incubator at 37 °C in the absence of CO2. After 3 h of incubation, cells were imaged in bright field using the Leica DMi8 microscope with 20X magnification. Images were quantified manually by counting the percentage of positive clusters.

### Irradiation

MCF-7 breast cancer cells were γ-irradiated with a total of amount of 10 gray (Gy) (1 Gy/min) using the YXLON X-ray apparatus (200 kv/4,0 mA – foc = 5,5). Lead plates on top of the chamber coverslips (Ibidi, 80826) were used to protect other cells from γ-radiation. After irradiation, the cells were cultured under normal circumstances for subsequently 24, 48, 72, or 96 h before they were fixed and stained for immunofluorescence.

### Reversible growth-arrest assay

D2.OR Fucci cells were cultured as described in the Matrigel-on-top section. Instead of a 48-well plate, the culture was upscaled to a 6-well plate. Cells were extracted from the Matrigel as described in the “FACS” section. After extraction, cells were plated in 2D in a μ-Slide 8 well (ibidi, 80826), and imaged on a Leica AF6000 LX with a Hamamatsu-C9100-02-COM4 camera and a HCX PL APO CS 20.0×0.75 DRY UV objective in 14-bit every 20 min for three days. Multiple positions were recorded and analyzed by ImageJ.

Alternatively, D2OR cells were cultured in the Matrigel-on-Top assay as described in the Matrigel-on-top section. About seven days after seeding, cells were extracted from Matrigel. Media was removed from the well and 200 μL of ice-cold trypsin was added. Mechanic disruption of the Matrigel was performed using P1000 tips and cycle of pipetting up and down. The suspension was then transferred to a 15-mL tube and incubated for 5 min at 37 °C. Again, mechanic disruption by pipetting up and down was performed using a P1000 and 10 mL of ice-cold PBS was added. Cells were centrifuged for 3 min at 1300 rpm and the supernatant was discarded. A second wash with PBS was performed before resuspending the pellet in 3D media. In total, 16 wells from a 48-well plate were pooled to reseed 1000 cells/well in 2D and 3D. Seeding was performed as described earlier in this section. Proliferation readout using Calcein-AM (see section Proliferation assay) was performed at day 1 and day 7 to determine the dormant/proliferative phenotype of the reseeded cells.

### Label retention

Vybrant DiI cell-labeling solution (5 µl/mL, V22888, Invitrogen) was directly added to cell suspensions at a density of 1 × 10^6^ cells/mL, and incubated for 7 min, followed by thorough rinsing with phosphate-buffered saline (PBS). Cells were seeded at 1 × 10^3^/well in a 48-well plate, and retention of the label was determined after eight days in culture using fluorescence-activated cell sorting (FACS) or microscopy. FACS experiments were carried out on a BD LSR IIFlowcytometer (BD Biosciences), and subsequently analyzed using BD FACSDiva software (BD Biosciences). To determine the label retention for each condition, cells were harvested after eight days of culture, washed, and analyzed by flow cytometry. Cells cultured in 3D were harvested from the basement-membrane matrix using Cell Recovery solution (Corning), in accordance with the manufacturer’s instructions. Cell morphology was observed by light microscopy, time-lapse images were captured by an AF6000 inverted wide-field microscope (Leica Microsystems), and label retention was quantified using confocal laser-scanning microscopy, for which the Leica SP5 was used with a 40x long working-distance objective, after which images were processed and analyzed using ImageJ Fiji software.

### Transcriptomic analysis

D2OR and D2A1 were seeded as described in the Cell culture section. In total, 10 wells of each cell line were pooled to be able to extract enough RNA. Cells were extracted from the Matrigel using the recovery solution from Corning. Briefly, media was removed and cells were washed three times with PBS. About 140uL of ice cold recovery solution was added per well. After 30 min of incubation on ice under agitation, the suspension (cells + Matrigel) was centrifuged at 1300 rpm for 3 min. RNA was extracted from the pellet using the Nucleospin kit (BioKé) protocol and sent to BGI for RNA sequencing using the Illumina-HiSeq2500/4000 sequencer.

Raw fastq files received from BGI were processed using the following pipeline. Raw data quality control was performed using FastQC v.0.11.4^47^. Adapter removal and trimming of low-quality reads was done with Trimmomatic v.0.32^48^, and afterward, a custom Python script (run with Python v.2.7.13) was used to remove reads with undetermined bases. Alignment of processed reads was performed using STAR v.2.5.3.a using GENCODE mouse release M15 (GRCm38)^49^. Aligned reads were then quantified using RSEM v.1.3.0^50^ and transcripts per million (TPM) were retrieved.

In order to identify genes showing interaction effect between cell lines and culture type, differential expression analysis was done using DESeq2 package for R software v.3.5.0^51^. Likelihood-ratio tests were done for each gene fitting a model adjusted for cell line, culture type, batch, and the interaction effect.

A preranked Gene Set Enrichment Analysis (GSEA, v2.2.07)^52^ was run with genes ranked using the negative logarithm (base 10) of the *p*-value and the sign of the beta coefficient from the differential expression model. For the interaction analysis, a positive-interaction beta coefficient denotes a higher slope in D2OR. This can correspond to multiple biological behaviors: upregulation in D2.OR 3D vs. 2D and downregulation in D2A1 3D vs. 2D, upregulation 3D vs. 2D in both D2OR and D2A1 but stronger in D2.OR, and downregulation 3D vs. 2D in both D2.OR and D2A1 but stronger downregulation in D2A1. Analogously, a negative-interaction beta coefficient corresponds to the opposite behaviors. A total of 4018 gene sets were tested after filtering for minimum set size of 30 genes, including specific sets to assess dormancy^53^ and senescence^54^, but also more general gene sets, like hallmarks, canonical pathways (KEGG, Biocarta, Reactome, and PID), and gene ontology, from MSigDB v.7.0^55^. For graphical representation, gene sets were clustered by gene overlap.

In order to assess intrinsic subtypes (PAM50) from the cell lines, we used Uniform Manifold Approximation and Projection (UMAP), a nonlinear dimension-reduction technique. To do so, we used primary breast cancer TCGA data (TPM downloaded from TCGA2BED)^56^ with available PAM50 information^57^ and computed a UMAP using a mouse-derived intrinsic subtype 1841-gene signature^58^, *n* = 50 neighbors and a minimal distance of 0.2.

### COBRA karyotyping

Metaphases from mouse cell lines were harvested using standard techniques, described in detail^59^. Multicolor fluorescence in situ hybridization (dubbed as COBRA-FISH) using mouse chromosome painting set to identify each chromosome was prepared and hybridized as presented in the detail published protocol^60^, following the procedure, including image acquisition and software tools.

### Animal experiments

All animal experimental protocols were approved by the animal welfare committee of the Leiden University Medical Center and the Dutch Animal Experiments Committee. Female BALB/c mice, aged between 8 and 12 weeks, were used exclusively.

Mice were injected with 1 × 10^6^ D2 H2B-Dendra2 cells loaded with DiI in the mesenteric vein. In short, mice received buprenorphine (0.01 mg/kg (Temgesic)) 30 min before surgery. Mice were anesthetized using injection anesthetics (70 mg/kg ketamine (anesketin) and 0.7 mg/kg medetomidine hydrochloride (sedastart) injected i.p.). Surgery was performed under aseptic conditions. A midline incision of ~1 cm was made in the belly region, the intestines were placed on a sterile gauze. Using a binocular, injection of tumor cells was performed in the mesenteric vein. Afterward, the puncture wound was closed by pressure using a q-tip, and the animal closed using a suture. After surgery, mice received 1 mg/kg atipamezole hydrochloride (antisedan) s.c. to wake up. About two weeks after injection, mice were sacrificed and the liver was harvested and fixed for one day in PLP-fixation mix (1% paraformaldehyde, 0.2% NaIO_4_, 61 mM Na_2_HPO_4_, 75 mM L-lysine and 14 mM NaH_2_PO_4_ in H_2_O). After fixation, tissues were incubated in 30% sucrose in PBS for >6 h, and frozen in tissue freezing medium (OCT). About 10-μm sections were obtained at three different heights of the liver (at least 250-μm apart). Sections were embedded in vectashield (hardset with DAPI), and imaged on the slide scanner (3DHISTECH panoramic 250).

### Intravital microscopy

Mice were injected with 1 × 10^6^ D2 H2B-Dendra2 cells loaded with DiI in the mesenteric vein. About 16 days later, the liver was surgically exposed and intravitally imaged on a Zeiss LSM 710 NLO upright multiphoton microscope equipped with a Mai Tai Deep See multiphoton laser (690–1040 nm) and a custom-fit stage with window holder. During imaging, the mice were anesthetized using isoflurane. Tumor cells visible underneath the window were recorded at multiple positions using z-stack timelapses. Images were collected in 8-bit and 512 × 512 pixels, with an xy-resolution of 0.25–083 μm and a z-resolution of 3 μm per pixel. Z stacks were recorded every 20 min for ~3 h. Dendra2 and DiI were excited with 960 nm, and emission was collected in BiG NDD (510–550 nm), BiG NDD (575–620). Timeseries were registered using the ImageJ FIJI plugin Descriptor-based series registration (2D/3D + t). Maximum projections were made of 2 or 3 Z planes. Some images were smoothed and gamma-corrected to enable visualization of lower-intensity cells.

### Scratch assay

In total, 25.000–50.000 D2.OR or D2A1 cells were plated in 96 wells plated in DMEM containing 10% FBS and pen/strep. The next day, wells were scratched according to Incucytes protocol, washed with PBS, and placed in serum-free DMEM. About 2–4 h after scratching the imaging started. At least seven wells per condition per experiment were imaged every 2 h. The scratch wound at the start of imaging was used to determine the relative percentage invasion of cells in that area over time. The wells were grouped to produce one number per experimental condition, then three experiments were combined for the analysis.

### Random-migration assay

D2.OR or D2A1 cells were plated on 0.2% gelatin-coated coverslips or on tissue culture-treated Ibidi μ-slides, and imaged at a temperature-controlled (37 C), humidified, CO_2_-controlled Leica AF6000 microscope for 19–24 h. Pictures were taken every 30 min–3 h, and were analyzed using the ImageJ Manual Tracking or Trackmate package.

### Invasion assay

In total, 5*10^4^ D2.OR and D2A1 cells (serum-starved 24 h) were plated in serum free medium on a Corning Biocoat Growth factor-reduced Matrigel invasion chambers (#354483). They were allowed to migrate toward a 10% FBS gradient in the lower compartment for 24 h. The Matrigel was removed, and the lower compartment stained with crystal violet and imaged with a 20x objective. The number of cells per field of view was quantified manually, and 10 fields of view per experiment were combined for quantification.

### Statistical analyses

Data were analyzed using software Graphpad Prism v5. Student’s *t*-test, Mann–Whitney U test, or one- or two-way ANOVA statistical tests were used where appropriate. Statistical significance was defined as n.s. *P* ≥ 0.05, *P ≤ 0.05, **P ≤ 0.001, ***P ≤ 0.001, ****P ≤ 0.0001. A post hoc analysis was only performed if a significant interaction was found. In the legend or figures, only significant analyses are shown, unless otherwise stated. Quantitative data are presented as the mean ± standard error of the mean, unless stated otherwise.

### Reporting summary

Further information on research design is available in the Nature Research Reporting Summary linked to this article.

## Supplementary information

Supplementary Data 1

Supplementary Data 2

Supplementary Information

Supplementary Movie 1

Supplementary Movie 2

Reporting Summary

## Data Availability

The sequencing data discussed in this publication have been deposited in NCBI’s Gene Expression Omnibus^61^ and are accessible through GEO Series accession number GSE172882. These are associated with Figs. 1**a**, 3**b–d**, supplementary figs. 1 and 9b.
