# Supplementary material for: Breast cancer dormancy is associated with a 4NG1 state and not senescence
Source: NPJ Breast Cancer. 2021 Oct 27;7:140. doi: 10.1038/s41523-021-00347-0 (PMC8551199; doi:10.1038/s41523-021-00347-0)
Supplement: Supplementary file 3 — Supplementary Information [file 41523_2021_347_MOESM3_ESM.pdf]

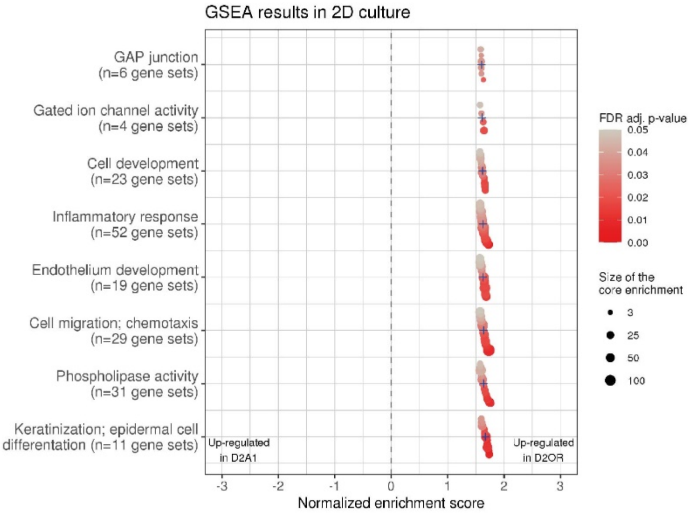

Supplementary Fig. 1: D2 cells are considered to be of the ER+/HER2+ basal subtype. GSEA analysis on the differential expression between D2A1 and D2.OR cultured in 2D. Gene-sets ordered by normalized enrichment score (NES) within each cluster. Blue crosses represent average NES in each cluster. Point color represents FDR-adjusted p-value and size represents core enrichment.

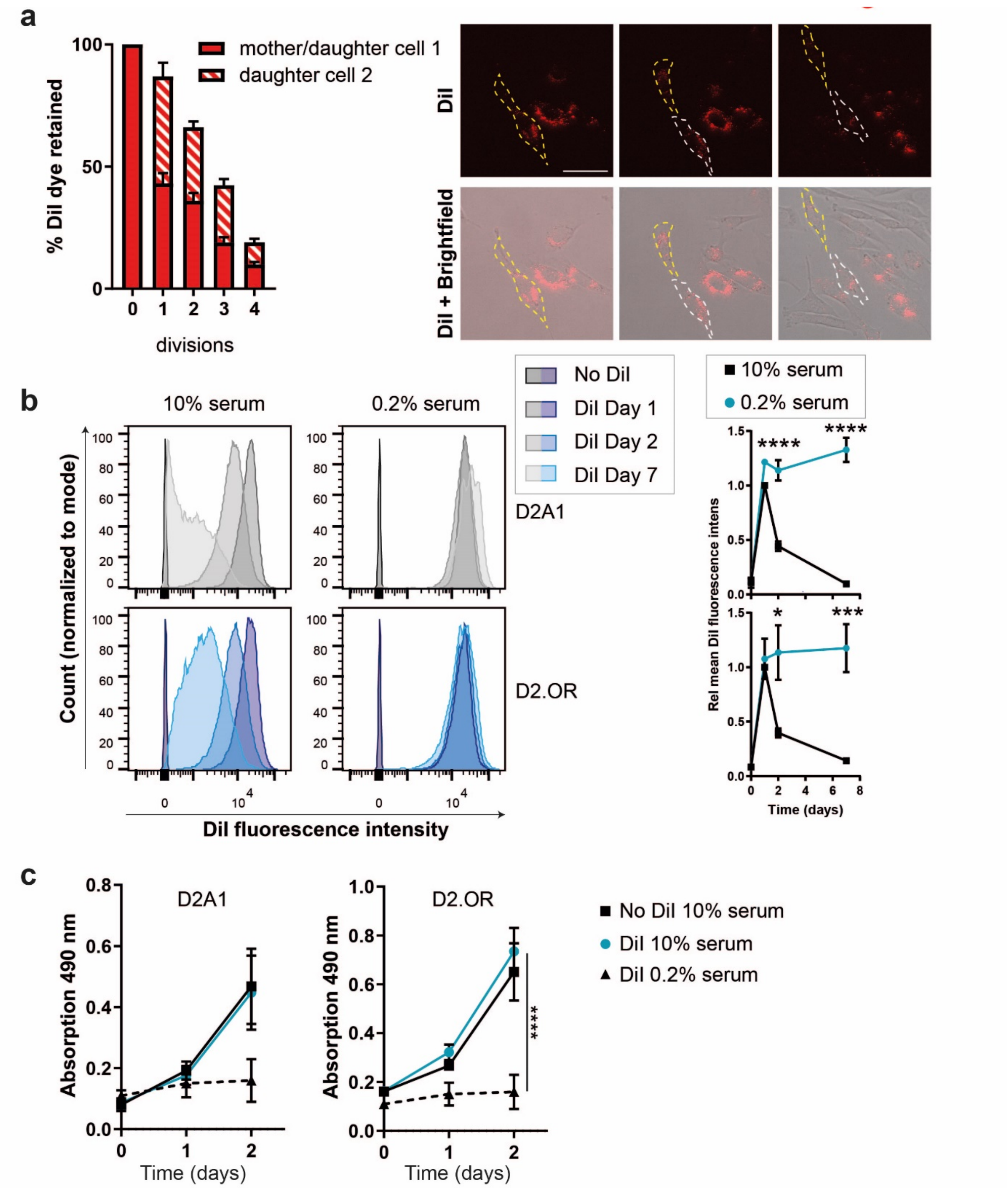

Supplementary Fig. 2: The effect of DiI loading of D2 cells. a Quantification (left) of live cell imaging time series (right) of D2A1 cells in 2D. Individual cells were tracked and total DiI fluorescence intensity per cell was determined (background subtracted) for mother cells (yellow dashed line) and subsequent daughter cells (white / yellow dashed line). N = 5 cells, scalebar, 50 μm. **b** FACS plots depicting D2 cells loaded with DiI and cultured (in 10 or 0.2% FBS) for 1, 2 or 7 days. Quantification on the right. N = 3. Interaction D2A1 (time x serum), F (3, 14) = 5.470. P = *. Interaction D2.OR (time x serum), F (3, 14) = 38.12, P = ****. **c** Quantification

of MTS assay of D2 cells loaded with or without DiI, cultured in 10% or 0.2% FBS for 0,1, and 2 days. N = 3. Interaction D2A1 (time x condition), $F(4,12) = 3.426$, P = *. Interaction D2.OR (time x condition) $F(4,12) = 5.144$, P = **.

P value was calculated using 2-way RM ANOVA. Error bars, s.e.m

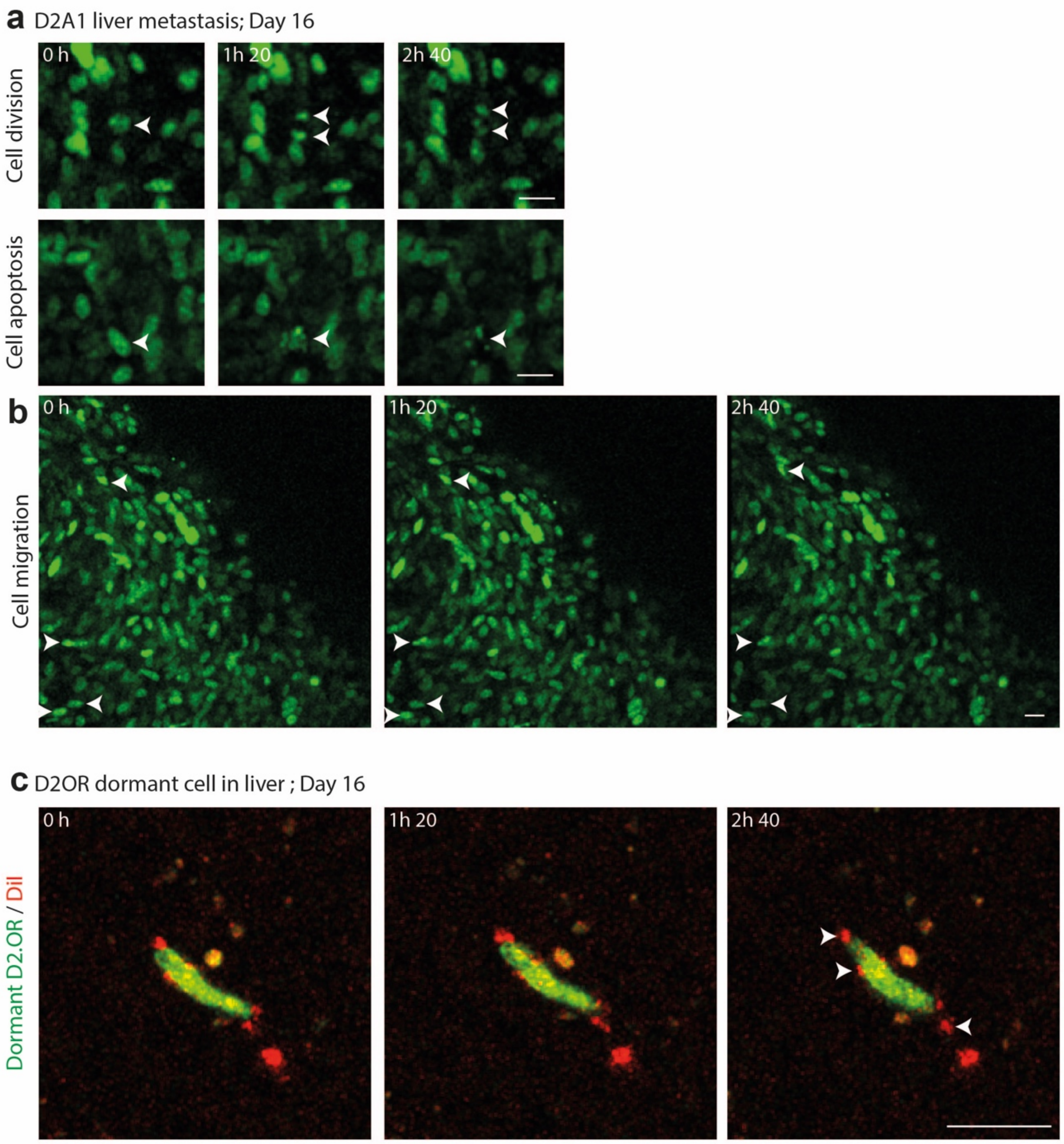

Supplementary Fig. 3: Intravital imaging of D2A1 and D2.OR cells in the liver. a-d D2A1 and D2.OR H2B-Dendra2 cells were intravitally imaged 16 days after injection in the mesenteric vein for ~3 hours. In D2A1 liver metastases cell division (a), apoptosis (a) and cell migration (b) were observed. D2.OR cells were loaded with DiI before injection, which was retained at day 16 (c). During 3-hour movies, the cells showed no signs of division, apoptosis or migration. Arrowheads indicate D2A1 cell division/apoptosis/migration or DiI dye in a D2.OR cell. Scalebars, 20 μm.

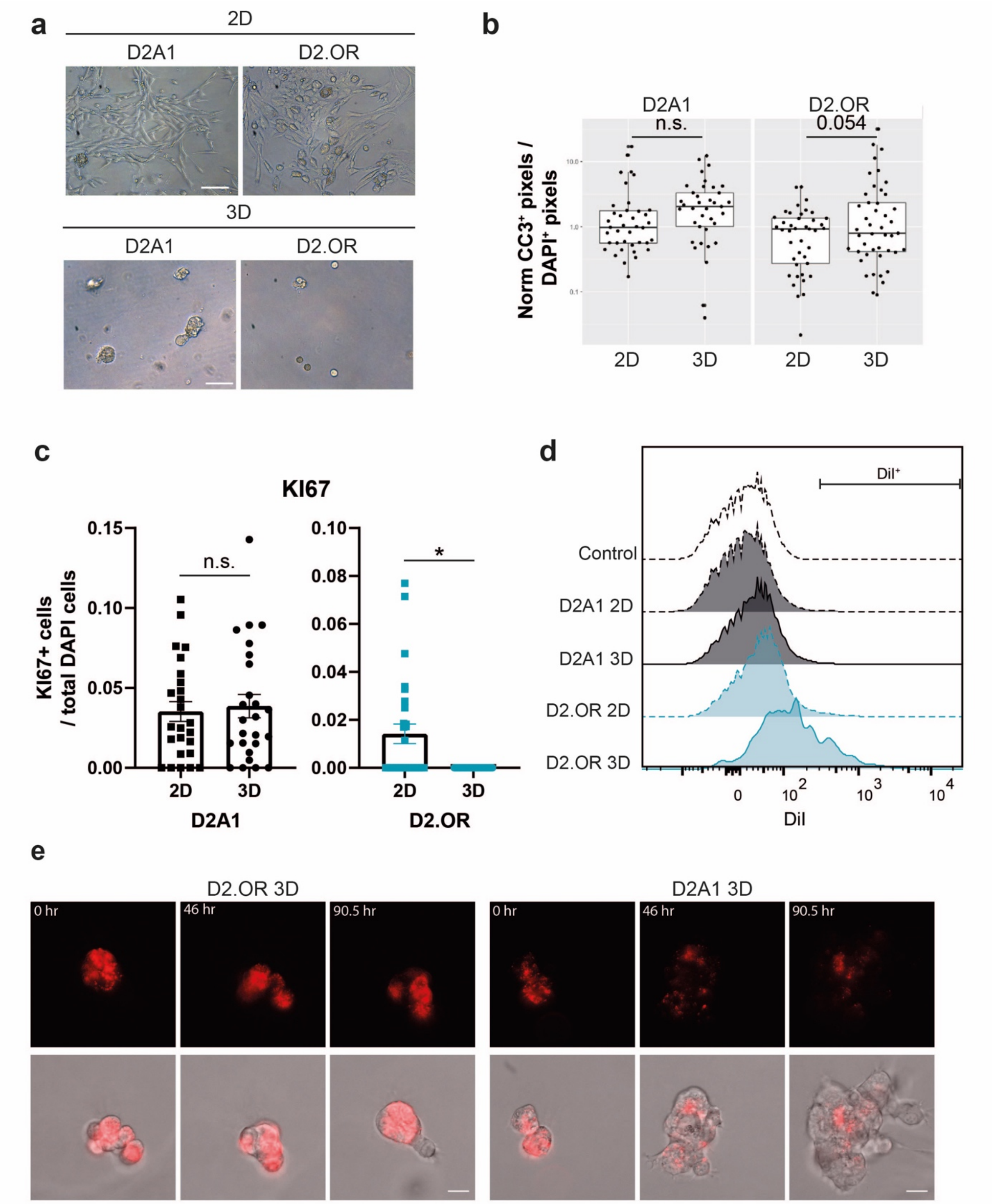

Supplementary Fig. 4: D2.OR cells are dormant in an *in vitro* 3D condition. a Images of D2 cells cultured in 2D or 3D for 6 days. Scalebar, 100 μm. **b** Quantification of immunostaining of D2 cells cultured for 4 days; per FOV cleaved caspase 3 (CC3)$^+$ pixels were divided to DAPI$^+$ pixels to account for the number of cells, and normalized per replicate to D2OR 2D. N ≥ 37 FOV in 4 replicates. Boxplot: median (centre line), 25/75 (box) and 5/95 (whiskers) percentile. **c** Quantification of

immunostaining of D2 cells cultured for 4 days; percentage of KI67+ cells per FOV. N = 25 FOV of 4 replicates. Error bar, s.e.m. **d** FACS plot related to figure **2b**. **e** Microscopy images from a time series of D2 cells loaded with DiI and grown in 3D for 4 days, imaged every 30 mins. Upper panels; DiI. Lower panels; DiI and brightfield. Scalebar, 20 μm. P values were calculated by 2-way ANOVA or nested t test.

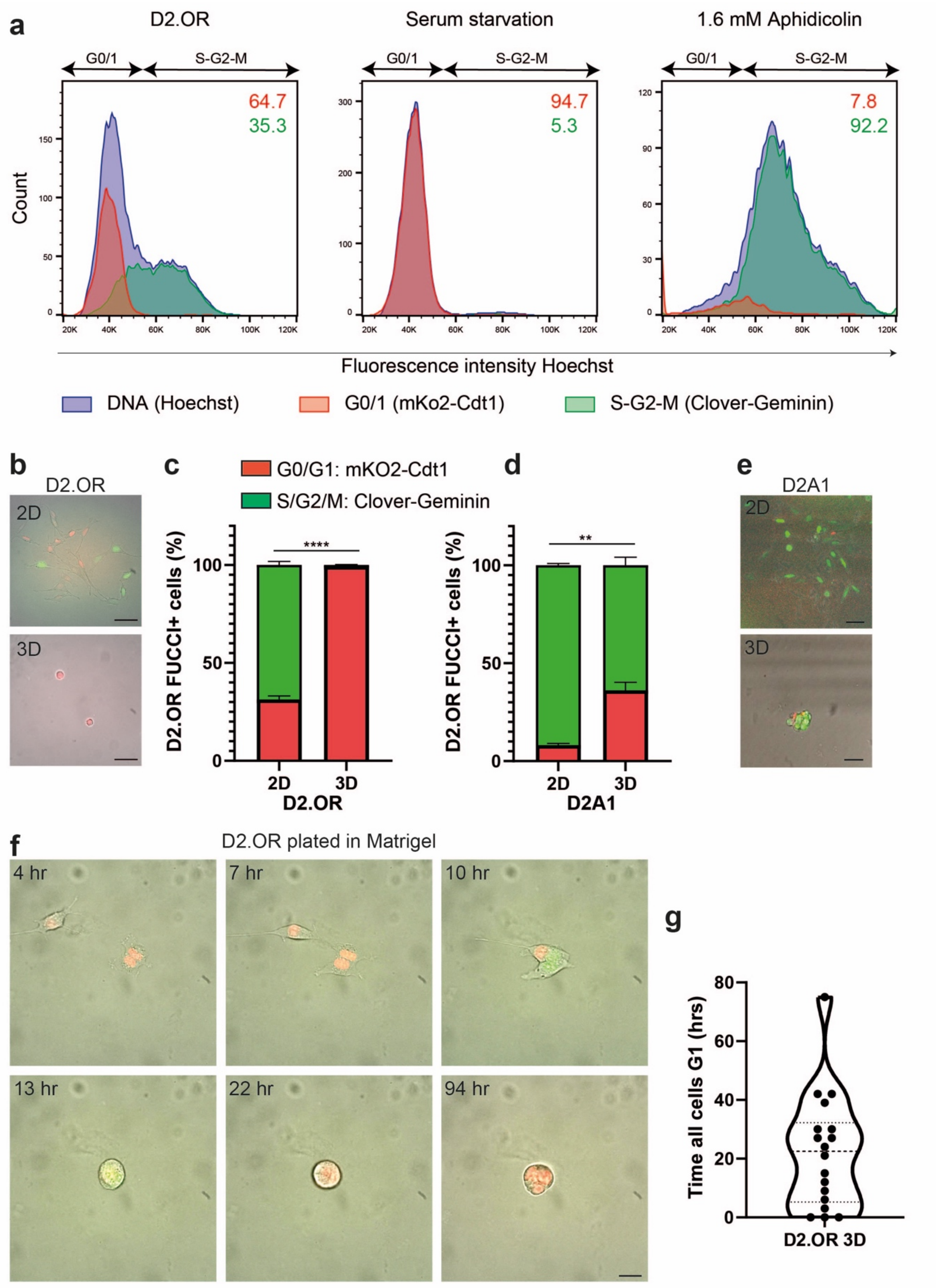

Supplementary Fig. 5: Characterization of D2.OR Fucci cells. a FACS plots depicting cell cycle status (based on Hoechst) of D2.OR cells in 2D treated with serum starvation (0.1% FBS) or aphidicolin [1.6mM] for 24 hrs. The percentage of mKo2 (red) or Clover (green) cells based on the Hoechst population is shown in the

top right corner. **b-e** Quantification of Fucci mko2^{+} or Clover^{+} D2.OR (c) or D2A1 (d) cells in microscopy images (b,e). D2.OR or D2A1 Fucci cells were cultured for 2 days in 2D and for 7 days in 3D. N = 3 tilescans in duplo. Scalebar, 50 μm. Error bar, s.e.m. **f** related to **Movie 2**. Images from time recordings of D2.OR Fucci cells plated in Matrigel. The time indicates the time after plating in Matrigel. Images were taken every 3 hours. Scalebar, 20 μm. **g** Related to **fig. 2d**. D2.OR Fucci cell clusters in 3D were analyzed in time lapse movies to determine the time until all cells in that cluster were mKo2^{+}. N = 18 clusters. P value was calculated by nested two-sided t test.

a

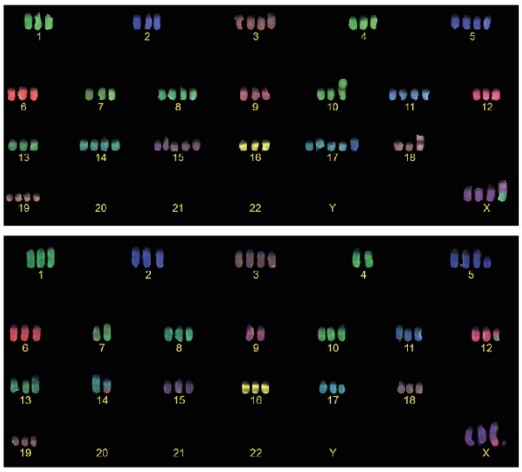

Cell line	Karyotype
D2.OR	68~71, XX der(X)t(X;8), +X, +3, +5, +8, rob(10;10)(q10;q10), +11, +14, +15, +15, der(17)t(2;17), rob(18;18)(q10;q10),+19
D2A1	56~61,XX,der(X)t(X;12),+mar(X), +3,+del(3),+del(5),der(7)t(4;7),-7,-9,-10, der(12)t(8;12),der(13)t(13;13), der(14)t(14;19),-14

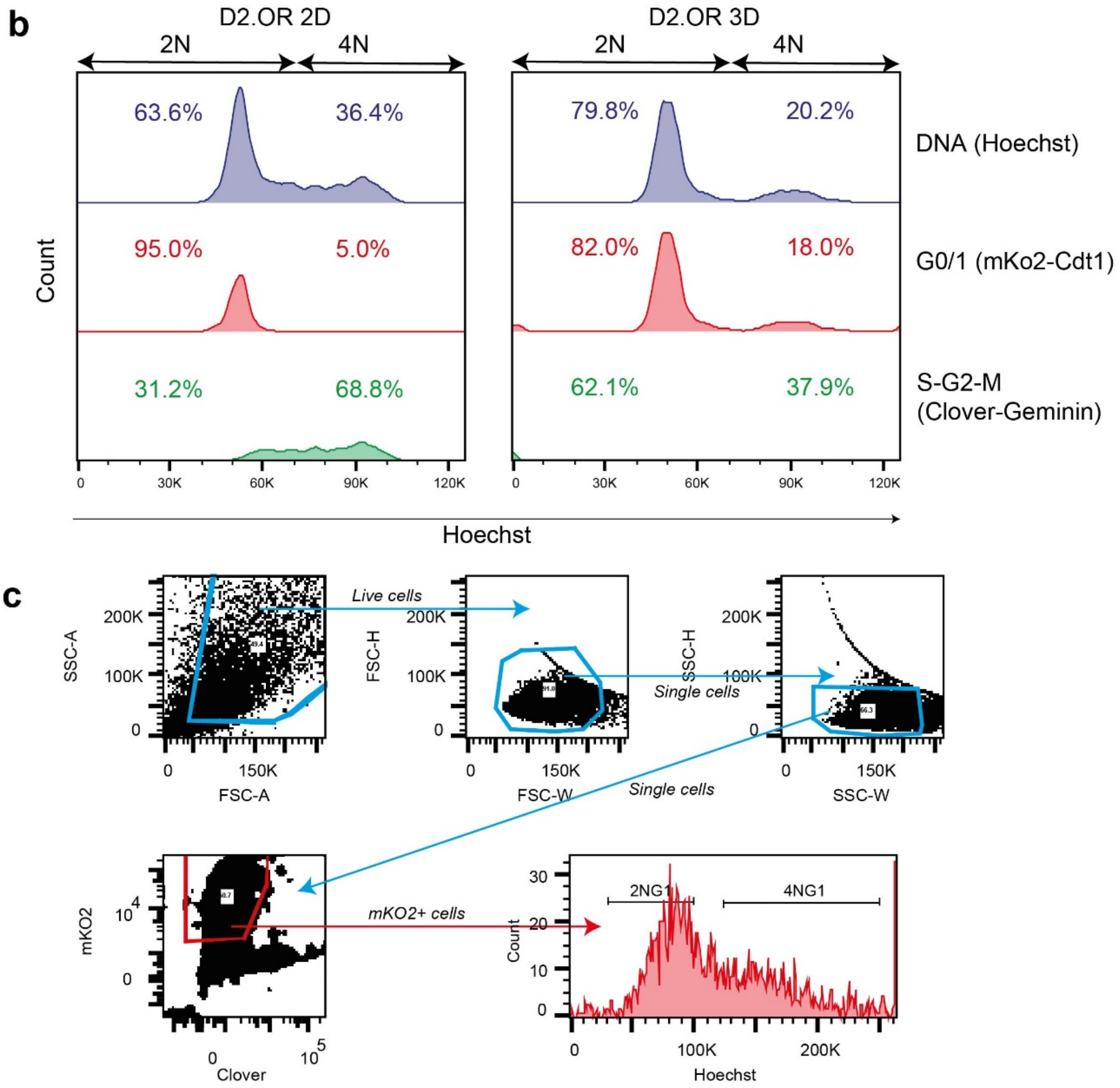

Supplementary Fig. 6: D2 cells are polyploid. a Cobra karyotyping of D2.OR and D2A1 cells in 2D. **b** FACS analysis related to **Fig. 2f**. Cell cycle analysis based on Hoechst is shown. Percentages indicate cells in 2N or 4N gate for that color. **c** related to **Fig. 2g-h**. FACs sorting and gating strategy for D2.OR cells grown in 3D

for 5 days and extracted from the Matrigel before subjected to sorting. The 2NG1 and 4NG1 gates were sorted, after which cells were plated on microscopy dishes and subjected to live cell microscopy for 5 days (imaging every 2 hours) to determine time until first division.

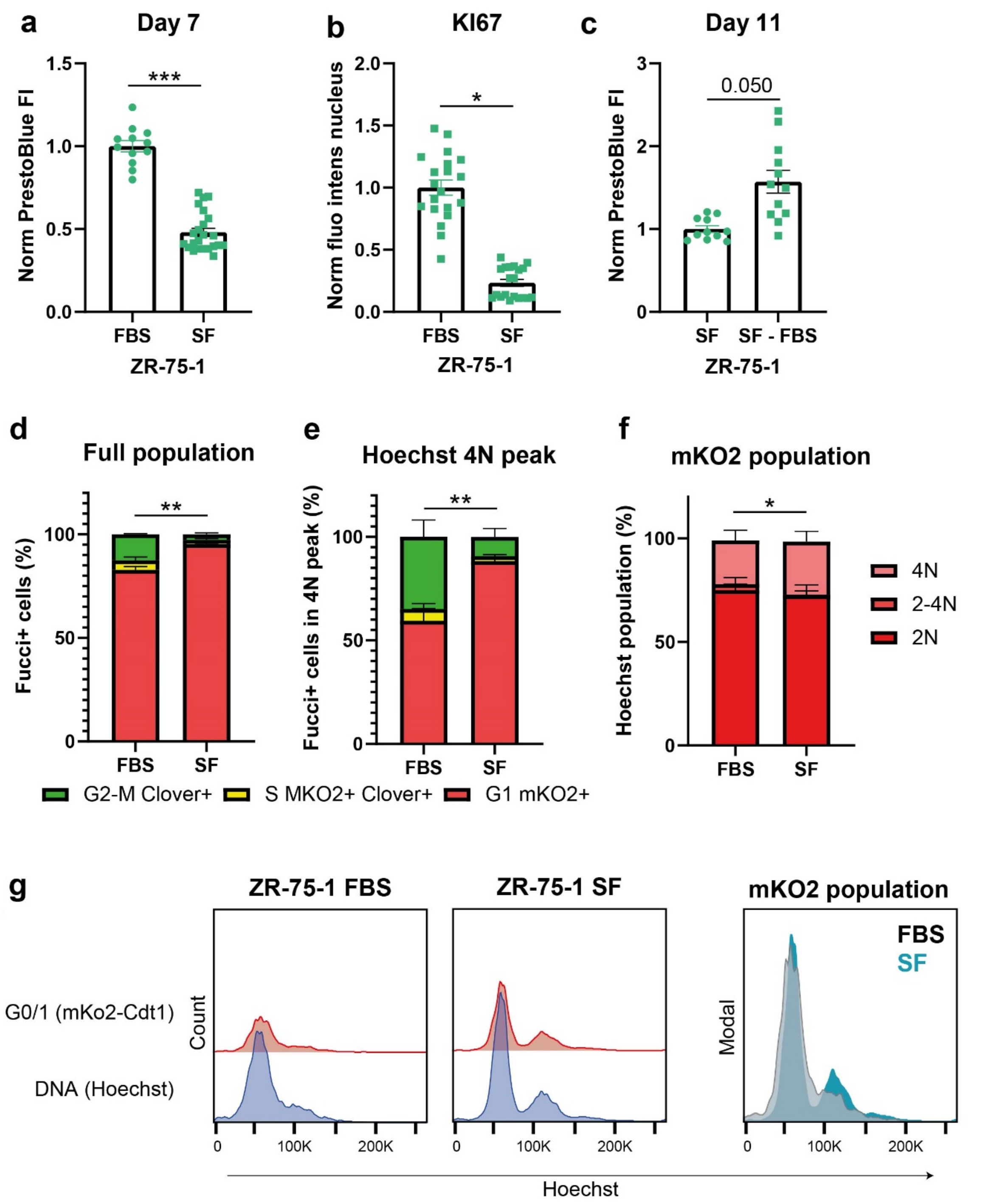

Supplementary Fig. 7: Characterization of the ZR-75-1 cell cycle arrest. a Proliferation analysis of ZR-75-1 cells in 10% FBS or serum free (SF) culture conditions in 2D, using PrestoBlue cell viability assay. N = 4 replicates in ≥triplo. **b** Quantification of nuclear KI67 staining intensity of ZR-75-1 cells cultured for 7 days in 10% FBS or SF conditions. Replicates normalized to FBS. N = 20 field of view (FOV) of 2 replicates. **c** Proliferation analysis of ZR-75-1 cells in 10% FBS or serum free (SF) culture conditions in 2D, using PrestoBlue cell viability assay. N = 4 replicates in

≥triplo/duplo. **d** Quantification of the percentage of ZR-75-1Fucci$^+$ cells (either Clover$^+$ or mKO2$^+$) after 7 days of culture by FACS. t test on mKO2 population. N= 3. **e** Quantification of the percentage of ZR-75-1Fucci$^+$ cells (either Clover$^+$ or mKO2$^+$, or double positive) in the Hoechst 4N gate after 7 days of culture by FACS. N = 3. T test on mKO2 population. **f** Quantification of FACS analysis showing the percentage of mKO2$^+$ ZR-75-1 Fucci+ cells containing 2N or 4N DNA (measured by Hoechst) after 7 days of culture. N = 3. T test on 4N population. **g** FACS plots showing the mKO2 and Hoechst profiles for ZR-75-1 cells. Related to d-f.
P values were calculated by two-sided paired T test or nested T tests. Error bars, s.e.m.

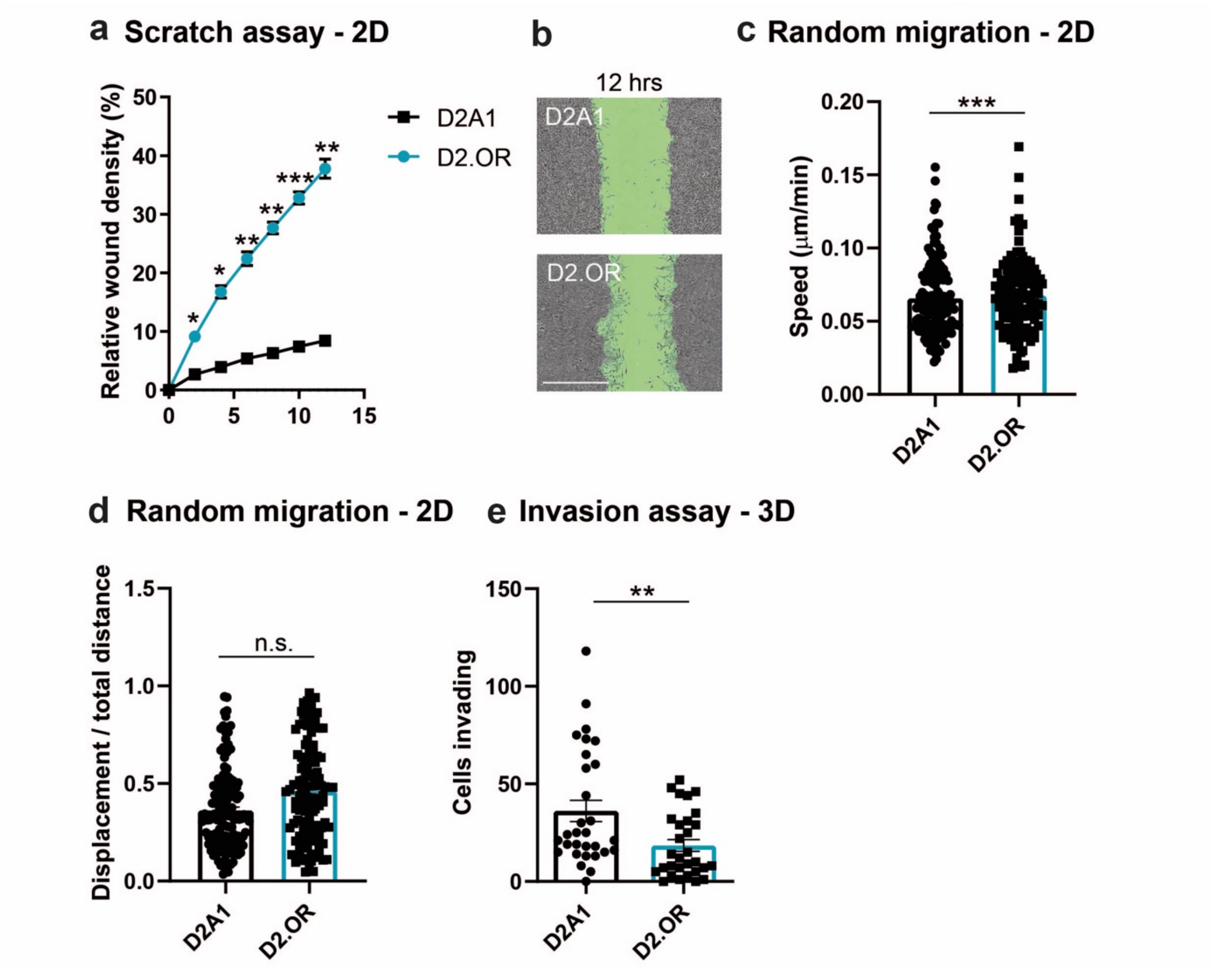

Supplementary Fig. 8: Characterization of D2 cell migration *in vitro*.

a Quantification of relative wound density 2-4 hours after scratching. **b** representative images (green, initial wound; grey, brightfield signal of the cells). Scalebar, μm. Time x cell line, F (6,24) = 237.2, P = ****. *Post hoc* test, D2A1 vs D2.OR various times indicated in figure. N = 3 with >7 repeats. P value was calculated using 2-way RM ANOVA. **c-d** Quantification of speed of cells (c) or displacement (d) in representative *in vitro* 2D random migration experiment, each dot represents a cell. Multiple cells per ≥ 3 fields of view were quantified. N = 3 experiments. **e** Quantification of number of cells that invaded a Matrigel-covered transwell in 24 hours. N = 3 experiments with each 10 fields of view.

P values were calculated by repeated measures ANOVA, or T tests. Error bars, s.e.m.

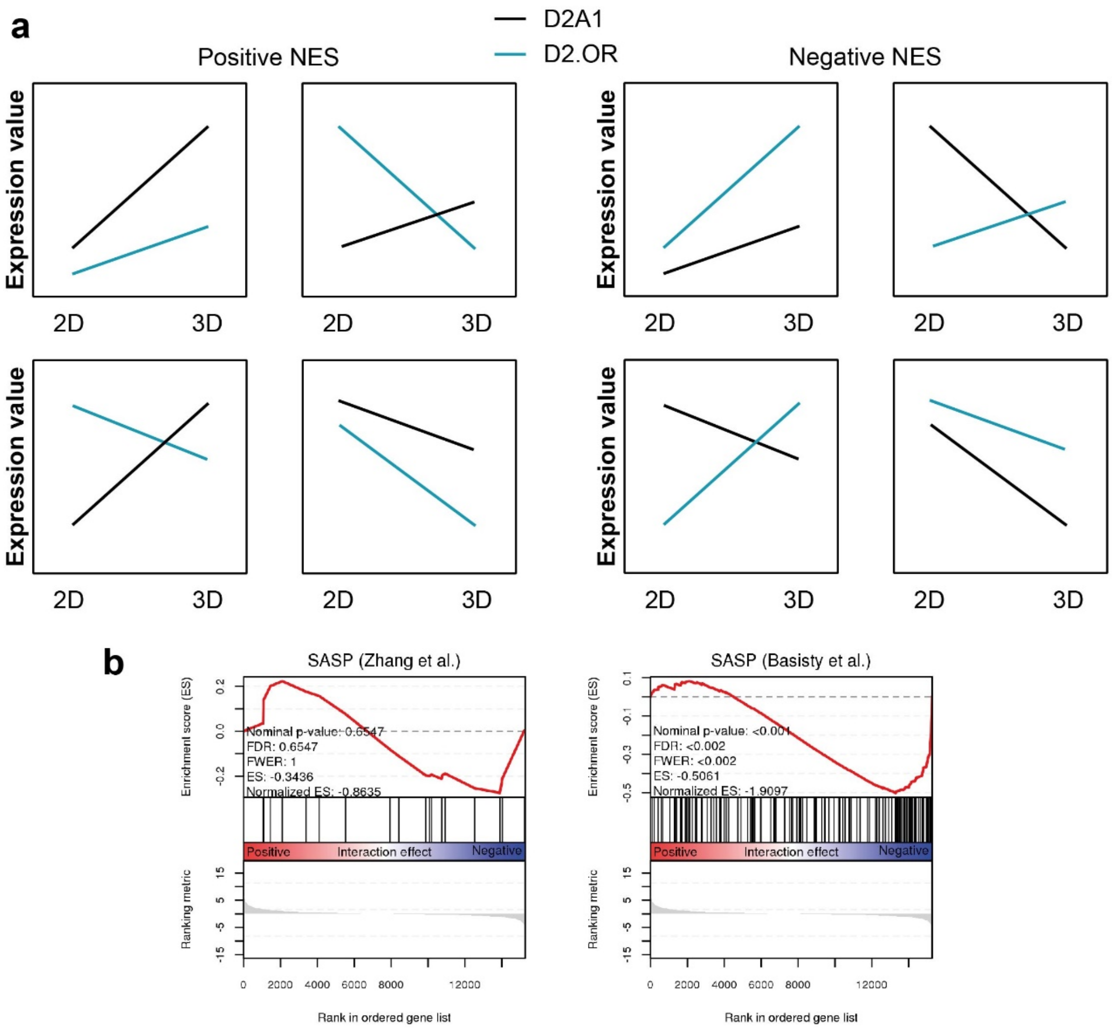

Gene set	Size	Enrichment score (ES)	Normalized ES (NES)	Nominal p	FDR	FWER
SASP_ZHANG	19	-0.34	-0.86	0.6547	0.6547	1
SASP_BASISTY	141	-0.51	-1.91	<0.001	<0.002	<0.002

Supplementary Fig. 9: Gene set enrichment analysis of D2 cells. a Related to **Fig 3b.** Cartoon explaining the interaction effects that result in a positive or negative normalized enrichment score (NES). **b** GSEA analysis of a published dormancy signature (Zhang *et al*) (**c**) or senescence signature (Basisty *et al*) (**d**) on the interaction effect when comparing D2A1 and D2.OR in 2D and 3D. ES, Enrichment score; RM, ranking metric.

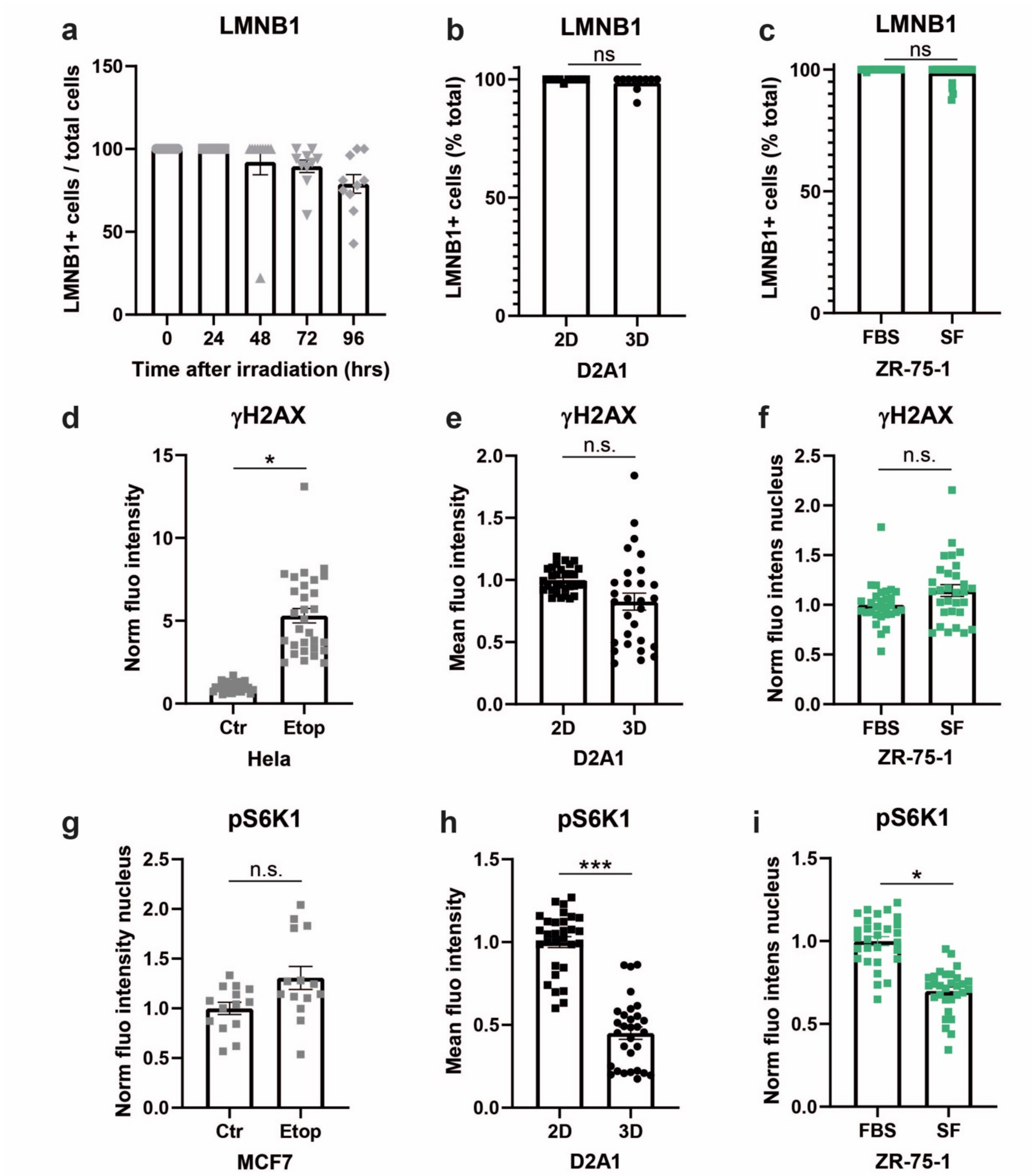

Supplementary Fig. 10: Characterization of senescence associated markers by immunofluorescence. a,d,g Senescence control, more details below. **b,e,h** D2A1 cells were cultured in 2D or 3D for 5 days and then subjected to immunofluorescence. Replicates normalized to 2D. N = 3, with 10 fields of view (FOV) per replicate. **c,f,i** ZR-75-1 cells were cultured in 2D for 7 days in 10% FBS or serum free (SF), and then subjected to immunofluorescence. Replicates normalized to FBS. N = 3, with 10 FOV per replicate. **a-c** Quantification of the percentage of indicated LMNB1+ cells in indicated culture conditions. **a** MCF7 cells cultured in 2D,

irradiated with 10 Gy (1Gy/min), and incubated for indicated time to induce senescence. Cells were then stained for DAPI and LaminB1. N = 10 FOV per condition. **d-f** Related to **Fig. 4c**. Quantification of nuclear γH2AX staining intensity of Hela cells cultured for 4 days in 2D. 24 hrs before fixation cells were treated with Etoposide (12.5 μM) to induce senescence. Replicates normalized to Hela. N = 32 field of view (FOV) of 3 replicates. **g-i** Left, Quantification of pS6K1 immunostaining of MCF7 cells cultured in 2D and treated with or without etoposide (12.5 μM) (senescence control). Replicates are normalized to MCF7 vehicle. N ≥ 10 field of view (FOV) of 2 replicates.
P value was calculated by nested T-test. Error bars, s.e.m.

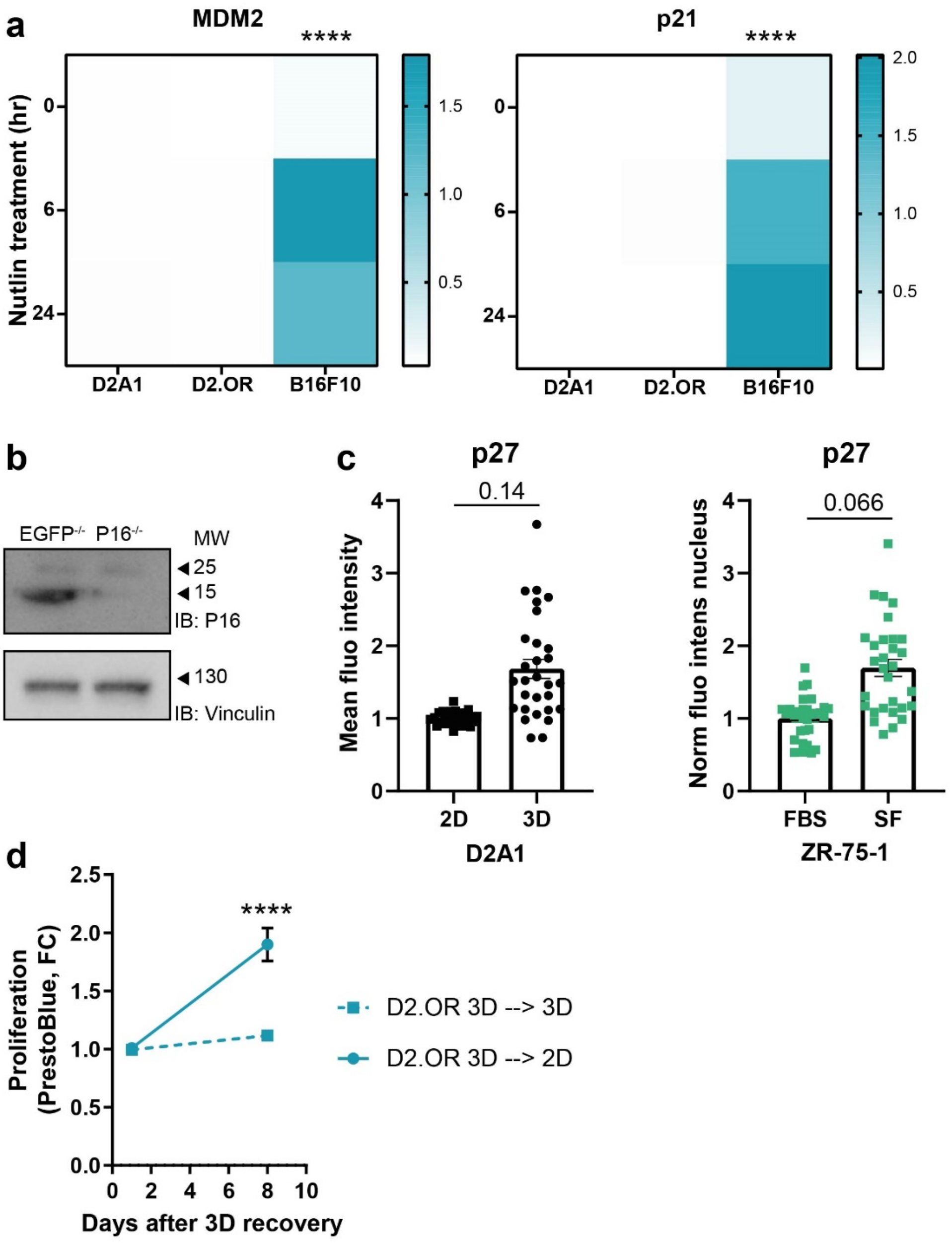

Supplementary Fig. 11: Characterization of dormancy and senescence parameters. a Heatmap showing qPCR normalized gene expression of D2A1, D2.OR and B16F10 (+ control) cells in 2D treated with Nutlin-3 [10 μM] for 0, 6 or 24 hours. Interaction MDM2, F (4,18) = 426.9, P = ****. *Post hoc* test, B16F10 vs D2.OR or D2A1, P = ****. Interaction p21, F (4,18) = 46.75, P = ****. *Post hoc* test, B16F10 vs D2.OR or D2A1, P = ****. **b** Immunoblot showing lysates of D2.OR EGFP-/- and D2.OR p16-/- cells cultured in 2D. **c** Immunofluorescence quantification of nuclear p27 protein in D2A1 (left) or ZR-75-1 (right). N = 3 replicates, with each 10 FOV per condition. **d** Quantification of D2.OR cell proliferation after extracting the cells from

3D and re-plating them into 2D or 3D. Time x cell, $F (1,6) = 30.24$, P = **. *Post hoc* test, 3D vs 2D day 8, P = ****. N = 2 in duplo.

P value was calculated by nested T-test or two way ANOVA test. Error bars, s.e.m.

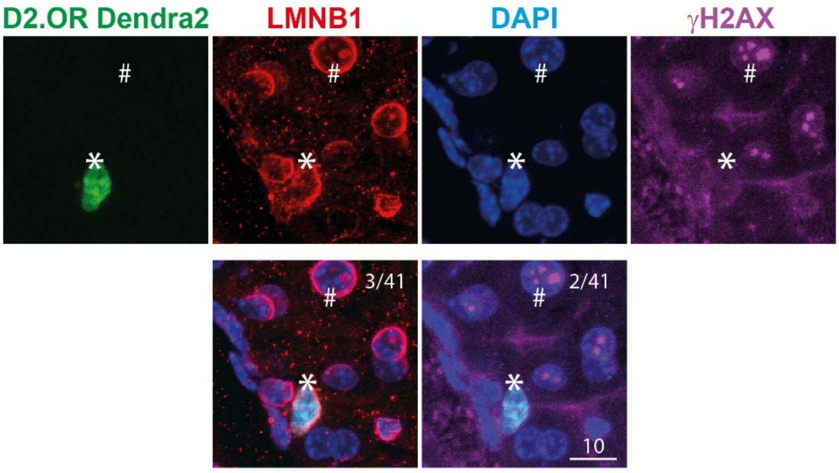

Supplementary Fig. 12: Characterization of senescence parameters *in vivo*. Microscopy images of immunohistochemistry on liver sections containing single (dormant) D2.OR tumor cells (related to **Fig 1e**). LMNB1 and γH2AX stainings were performed. Up; individual stains are indicated. Below; merge images are indicated. * indicates dormant D2.OR cell, and # indicates hepatocyte (positive control for staining). The numbers at the upper right corner indicate the amount of LMNB1+ or γH2AX- single D2.OR cells were present in the liver sections. Scalebar, 10 μm.

Related to Supplementary Fig 11b

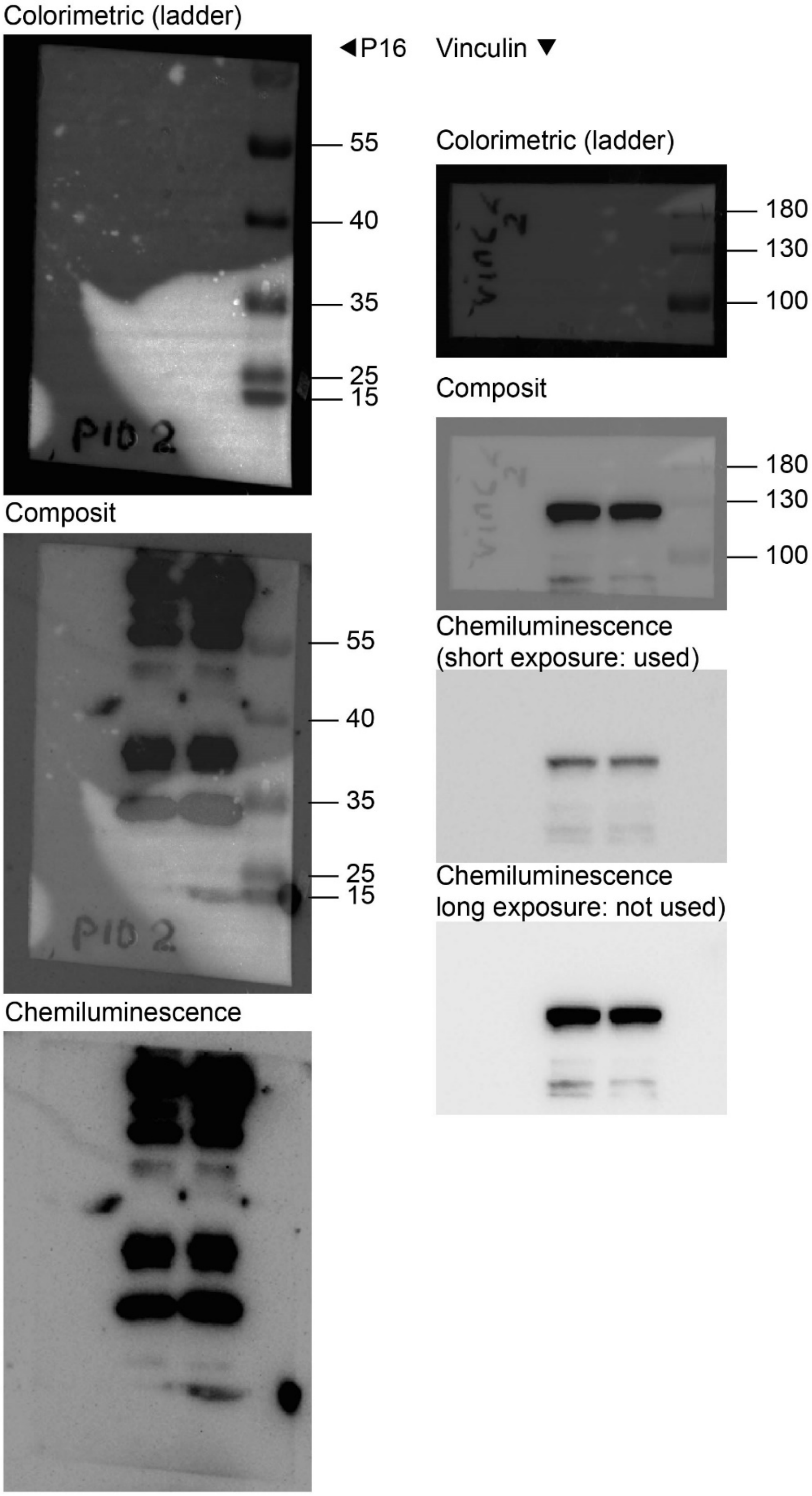

Supplementary Fig. 13: Uncropped westernblot images related to Supplementary Fig 11b.

Related to Fig 4e

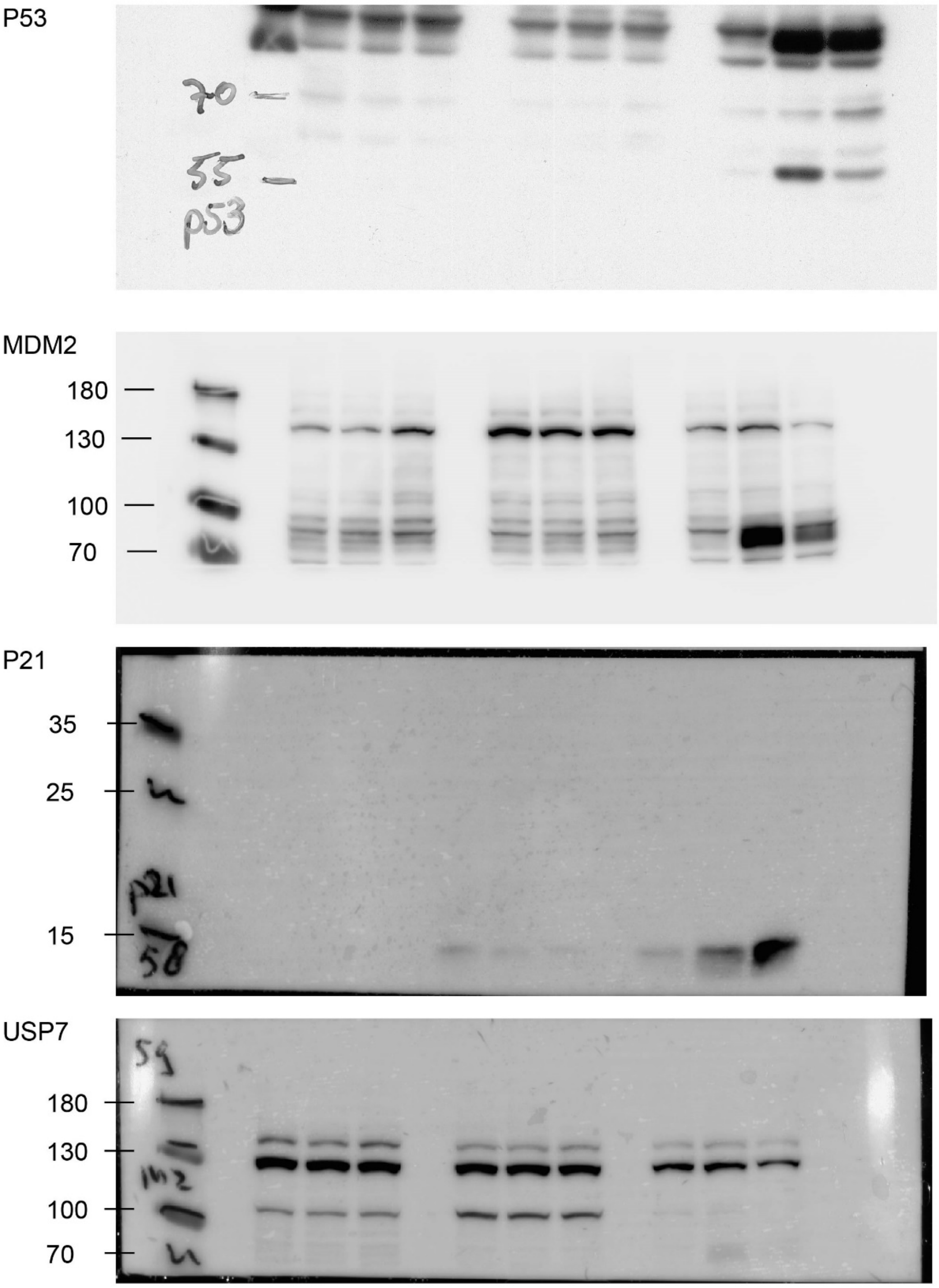

Supplementary Fig. 14: Uncropped westernblot images related to Supplementary Fig 4e.

Movie 1: Live cell imaging of D2.OR Fucci cells plated in Matrigel. D2.OR Fucci cells were plated in Matrigel. Time indicates time after plating. Images are a merge of brightfield, Fucci mKO2 (Red) and Clover (Green) taken every 3 hours.

Movie 2: Live cell imaging of dormant D2.OR Fucci cells extracted from Matrigel and replated in 2D. D2.OR Fucci cells were cultured in Matrigel for 4 days. Then, the cells were extracted from the Matrigel and replated on plastic μ-ibidi dishes and imaged every 20 minutes for 3 days.

Supplementary table 1. Gene sets included in GSEA analysis comparing D2OR and D2A1 in 2D.

Supplementary table 2. Gene sets included in GSEA analysis comparing the interaction effect when comparing D2A1 and D2.OR in 2D and 3D.
