# Supplementary material for: Breast cancer dormancy is associated with a 4NG1 state and not senescence
Source: NPJ Breast Cancer. 2021 Oct 27;7:140. doi: 10.1038/s41523-021-00347-0 (PMC8551199; doi:10.1038/s41523-021-00347-0)
Supplement: Supplementary file 6 — Reporting Summary [file 41523_2021_347_MOESM6_ESM.pdf]

nature research

Corresponding author(s): Laila Ritsma

Last updated by author(s): Aug 11, 2021

Reporting Summary

Nature Research wishes to improve the reproducibility of the work that we publish. This form provides structure for consistency and transparency in reporting. For further information on Nature Research policies, see our Editorial Policies and the Editorial Policy Checklist.

Statistics

For all statistical analyses, confirm that the following items are present in the figure legend, table legend, main text, or Methods section.

n/a	Confirmed	
☐	☒	The exact sample size (*n*) for each experimental group/condition, given as a discrete number and unit of measurement
☐	☒	A statement on whether measurements were taken from distinct samples or whether the same sample was measured repeatedly
☐	☒	The statistical test(s) used AND whether they are one- or two-sided<br>*Only common tests should be described solely by name; describe more complex techniques in the Methods section.*
☒	☐	A description of all covariates tested
☐	☒	A description of any assumptions or corrections, such as tests of normality and adjustment for multiple comparisons
☒	☐	A full description of the statistical parameters including central tendency (e.g. means) or other basic estimates (e.g. regression coefficient) AND variation (e.g. standard deviation) or associated estimates of uncertainty (e.g. confidence intervals)
☐	☒	For null hypothesis testing, the test statistic (e.g. *F*, *t*, *r*) with confidence intervals, effect sizes, degrees of freedom and *P* value noted<br>*Give P values as exact values whenever suitable.*
☒	☐	For Bayesian analysis, information on the choice of priors and Markov chain Monte Carlo settings
☒	☐	For hierarchical and complex designs, identification of the appropriate level for tests and full reporting of outcomes
☒	☐	Estimates of effect sizes (e.g. Cohen's *d*, Pearson's *r*), indicating how they were calculated

Our web collection on statistics for biologists contains articles on many of the points above.

Software and code

Policy information about availability of computer code

Data collection	No software was used for data collection
Data analysis	FastQC v.0.11.4, Trimmomatic v.0.32, Python v.2.7.13, STAR v.2.5.3.a, GENCODE mouse release M15 (GRCm38), RSEM v.1.3.0, DESeq2 package for R software v.3.5.0, GSEA, v2.2.07, MSigDB v.7.0

For manuscripts utilizing custom algorithms or software that are central to the research but not yet described in published literature, software must be made available to editors and reviewers. We strongly encourage code deposition in a community repository (e.g. GitHub). See the Nature Research guidelines for submitting code & software for further information.

Data

Policy information about availability of data

All manuscripts must include a data availability statement. This statement should provide the following information, where applicable:

- Accession codes, unique identifiers, or web links for publicly available datasets
- A list of figures that have associated raw data
- A description of any restrictions on data availability

The sequencing data discussed in this publication have been deposited in NCBI's Gene Expression Omnibus (Edgar et al., 2002) and are accessible through GEO Series accession number GSE172882 (https://www.ncbi.nlm.nih.gov/geo/query/acc.cgi?acc=GSE172882). These are associated with figures 1a, 3b-d, supplementary figures 1 and 9b.

Field-specific reporting

Please select the one below that is the best fit for your research. If you are not sure, read the appropriate sections before making your selection.

☒ Life sciences ☐ Behavioural & social sciences ☐ Ecological, evolutionary & environmental sciences

For a reference copy of the document with all sections, see nature.com/documents/nr-reporting-summary-flat.pdf

Life sciences study design

All studies must disclose on these points even when the disclosure is negative.

Sample size	For mice experiments: using pilot experiments it was determined that the coefficient of variation was <25%, suggesting that 5 animals would be enough (based on similar experiments done by others with a similar variation: Eckhardt, Mol Canc Res, 2005 / Johnstone, Dis Mod Mech, 2015). To account for potential animal loss, we included 1 extra animal per group.
Data exclusions	No data were excluded from the results.
Replication	All findings have been replicated in at least 3 biological repeats, unless otherwise mentioned in the figure legend. If technical repeats were taken along, it is indicated in the figure legend.
Randomization	Mice were randomly taken from their cage and injected with the two different cell lines.
Blinding	While sacrificing the animals, we were blinded for the cells that were injected as the mice were given a random number.<br>Quantification of the sections for determination of the hepatic replacement area was also done in a blinded manner based on the animal numbers.

Reporting for specific materials, systems and methods

We require information from authors about some types of materials, experimental systems and methods used in many studies. Here, indicate whether each material, system or method listed is relevant to your study. If you are not sure if a list item applies to your research, read the appropriate section before selecting a response.

Materials & experimental systems

n/a	Involved in the study	
☐	☒	Antibodies
☐	☒	Eukaryotic cell lines
☒	☐	Palaeontology and archaeology
☐	☒	Animals and other organisms
☒	☐	Human research participants
☒	☐	Clinical data
☒	☐	Dual use research of concern

Methods

n/a	Involved in the study	
☒	☐	ChIP-seq
☐	☒	Flow cytometry
☒	☐	MRI-based neuroimaging

Antibodies

Antibodies used	Ki67 (ab16667, AbCam, 1:200), Cleaved-Caspase3 (9661, cell signaling Technology, 1:200), Anti-phospho-Histone H2A.X (Ser139) (clone JBW301, 05-636 Millipore, 1:1000), p-p70 S6 kinase (pS6K1)(Santa cruz, sc-8416, 1:100), LMNB1 (abcam, ab16048, 1:500), p27 (3698, Cell signaling technology, 1:800).<br>Anti-CDKN2A/p16INK4a ([EPR20418] (Abcam, ab211542)), Anti-Vinculin (hVIN-1 (Sigma Aldrich, V9131, 1:1000)), anti-USP7 (Bethyl Laboratories, A300-033A), anti-MDM2, clone 3G9 (Merck Millipore, 04-1530), anti-p53 clone 1C12 (Cell Signaling Technology), anti-p21, Clone F5 (Santa Cruz Biotechnology).
Validation	All the used antibodies are commercially available and have been validated by the following manufactures: Millipore, Sigma-Aldrich, Cell Signalling Technology, Abcam, Bethyl Laboratories and Santa Cruz. Validation reports can be found on their websites using the catalog number indicated above. In addition, positive controls were taken along for Anti-phospho-Histone H2A.X (Hela cells treated with Etoposide), KI67 (D2.OR cells growing in full serum in 2D), and a negative controls for LMNB1 (MCF7 cells Gamma irradiated --> become senescent and loose LMNB1).

Eukaryotic cell lines

Policy information about cell lines

Cell line source(s)	Mouse mammary carcinoma cell lines D2A1 and D2.OR were obtained from Karmanos Cancer Institute (F.R. Miller)<br>Human breast cancer cells ZR-75-1 (in house)

	Mouse embryonic fibroblast (MEF) cells (in house)<br>Mouse melanoma cancer cells B16F10 (in house)<br>Human breast cancer cells MCF-7 (in house)<br>Human cancer cells Hela (in house)<br>Human kidney cells HEK293T (in house)
Authentication	None of the cell lines were authenticated
Mycoplasma contamination	All cell lines were routinely (bi-monthly) tested negative for mycoplasma
Commonly misidentified lines<br>(See ICLAC register)	NA

Animals and other organisms

Policy information about studies involving animals; ARRIVE guidelines recommended for reporting animal research

Laboratory animals	Female BALB/c mice, aged between 8 and 12 weeks, were used exclusively.
Wild animals	NA
Field-collected samples	NA
Ethics oversight	All animal experimental protocols were approved by the animal welfare committee of the Leiden University Medical Center and the Dutch Animal Experiments Committee.

Note that full information on the approval of the study protocol must also be provided in the manuscript.

Flow Cytometry

Plots

Confirm that:

- [x] The axis labels state the marker and fluorochrome used (e.g. CD4-FITC).
- [x] The axis scales are clearly visible. Include numbers along axes only for bottom left plot of group (a 'group' is an analysis of identical markers).
- [x] All plots are contour plots with outliers or pseudocolor plots.
- [x] A numerical value for number of cells or percentage (with statistics) is provided.

Methodology

Sample preparation	2D: Cells were collected and diluted to 1x10^6 cells/mL suspension in 0.2% BSA in PBS. Thereafter, 2x10^6 cells were incubated with Hoechst 33342 (1 mg/mL) (Thermofisher Scientific, 62249) to stain the nuclei. After incubation in the dark for 30 minutes at 37°C, cells were collected in 200-500 µL PBS and subjected to flow cytometry.<br>3D condition: Cells were stained with Hoechst 33342 (1 mg/mL) (Thermofisher Scientific, 62249) for 30 minutes at 37°C in their dish. Thereafter, cells were extracted from Matrigel according to our self-optimized protocol in which every step is performed on ice and all tips/pipets/tubes are pre-coated with 2,5% BSA (Sigma Aldrich) in PBS to yield the highest number of living cells. The cells were washed two times with PBS, ice-cold Cell Recovery Solution (Corning) was added and cells were gently rocked 20 to 30 minutes on ice in order to dissolve the Matrigel. After dissolvement was confirmed by microscopy, 1% BSA (Sigma Aldrich) was added to the cell suspension and cells were centrifuged (twice) (approximately 280g at 4°C) for 5 minutes. After discarding the supernatant, the cells were resuspended in 200-500µL 0.5% BSA (Sigma Aldrich) (and 1mM EDTA) in PBS and subjected to flow cytometry.
Instrument	Flow cytometry was performed on the BD LSR II machine and BD FACSDiva software, and sorts were performed on BD FACS Aria machines.
Software	Recordings were analysed using the FlowJo V10 software.
Cell population abundance	-
Gating strategy	For all sorts and analyses, gating started by selecting live cells using a SSC-A/FSC-A gate. Then single cells were identified using a FSC-A/FSC-H gate. mKO2 (PE-A), Clover (Alexa Fluor 488-A), and Hoechst (Pacific Blue-A) were then used to identify indicated populations. A gating example is given in Supplementary Figure S6c.

- [x] Tick this box to confirm that a figure exemplifying the gating strategy is provided in the Supplementary Information.
